# Safety Assessment of Herbal Food Supplements: Elemental Profiling and Associated Risk

**DOI:** 10.3390/foods12142746

**Published:** 2023-07-19

**Authors:** Ljilja Torović, Slađana Vojvodić, Danijela Lukić, Branislava Srđenović Čonić, Sanja Bijelović

**Affiliations:** 1 Department of Pharmacy, Faculty of Medicine, University of Novi Sad, Hajduk Veljkova 3, 21000 Novi Sad, Serbia; sladja.vojvodic@uns.ac.rs (S.V.); branislava.srdjenovic-conic@mf.uns.ac.rs (B.S.Č.); 2 Center for Medical and Pharmaceutical Investigations and Quality Control, Faculty of Medicine, University of Novi Sad, Hajduk Veljkova 3, 21000 Novi Sad, Serbia; 3 Institute of Public Health of Vojvodina, Futoška 121, 21000 Novi Sad, Serbia; danijela.lukic@izjzv.org.rs (D.L.); sanja.bijelovic@mf.uns.ac.rs (S.B.); 4 Faculty of Medicine, University of Novi Sad, Hajduk Veljkova 3, 21000 Novi Sad, Serbia

**Keywords:** risk assessment, hazard quotient, hazard index, margin of exposure, lifetime cancer risk

## Abstract

Increased usage of herbal food supplements by both the paediatric and adult populations prompted a health risk assessment study. The elemental profiles of 52 supplements collected in Serbia were obtained using ICP-MS. The calculated contribution to permitted daily exposure was in compliance with the guidelines for elemental impurities: up to 46.8% for Pb (infants), 67.2% for Cu (toddlers), 6.8% for As (preschool children), and 8.0% for Hg (adolescents). Hazard quotients indicated high exposure of toddlers to Cu (100.9%) and Zn (112.7%), although by only one supplement, for which hazard indices ranged from 229 to 105% (aligning from toddlers to adolescents). Based on the margin of exposure, As and Pb were not of safety concern. Conversely, the proportion of supplements exceeding the acceptable level of lifetime cancer risk due to As exposure varied from 41.9% in adolescents to 54.3% in adults. Simultaneous use of multiple supplements over a prolonged period of time could further deepen health concerns.

## 1. Introduction

The use of food supplements has increased worldwide during the last few decades, with about 50–75% of the population consuming them occasionally and half of them regularly [1]. According to the EU Directive 2002/46, food supplements’ means “foodstuffs the purpose of which is to supplement the normal diet and which are concentrated sources of nutrients or other substances with a nutritional or physiological effect, alone or in combination, marketed in dose form, namely forms such as capsules, pastilles, tablets, pills, and other similar forms, sachets of powder, ampoules of liquids, drop dispensing bottles, and other similar forms of liquids and powders designed to be taken in measured small unit quantities” [2]. The European legislative framework requires certain particulars related to supplement labeling, including a statement about the recommended daily dose and a warning to consumers not to exceed it [2]. The use of primarily vitamins and various herbal products has additionally increased during the COVID-19 pandemic, both due to the overload of the health systems and due to people turning to pharmacies as the most accessible primary health care [3]. The widespread belief that herbal products can help in the prevention and treatment of various diseases is the reason for the upward trend in the use of food supplements [1,4,5]. Food supplements are readily available on the market and could be taken without a doctor’s prescription, often claiming to strengthen the immune system, support physiological processes in the organism, and fight against various conditions/diseases. This is additionally supported by massive marketing campaigns/promotions. Most consumers believe that natural products do not pose any health risks and that they have to be safe if they are already available on the market/over the counter [6]. However, the legislation on food supplements is not as strict as the legislation on conventional medicines, and that directly affects the safety of the market supply.

Due to their inherent toxicity, a wide range of elemental impurities potentially present in supplements could be of great concern to consumers’ health [7]. Elemental impurities might even catalyze the decomposition of supplements’ active ingredients [8]. The impurities may originate from the raw materials, the environment where the plant was grown, contamination of agricultural soils (especially in industrialized and high-intensive traffic areas), irrigation waters, or the atmosphere, as well as the manufacturing equipment, processing, and packaging materials [8,9,10]. Plants uptake elements through the air, water, and soil, and these elements may remain in the processed product [11]. Soil may be contaminated with atmospheric toxic metals, irrigation water, automobile and industrial exhausts, pesticides, and fertilizers [12]. Elemental impurities may also be a consequence of the intentional addition of specific elements during the manufacturing process, such as catalysts. Other sources of contamination could be materials used in equipment or on surfaces that are in direct contact with raw materials or the finished product. Corrosion or leakage may also lead to the incorporation of elemental impurities into the finished product, but generally, raw materials are the most common sources of contamination [13]. Additional sources of elemental impurities may be water, which is used in most of the processes, or elements could be added as active ingredients in order to improve their effects on human health [13].

Several recent research studies investigated the factors associated with elemental contamination of food supplements. Statistically significant positive associations were found between Cd and As contamination and supplements containing herbs (in comparison with mineral-vitamin supplements) as well as supplements in liquid/powder and soft-gel dosage forms (in comparison with tablets, capsules, and caplets) [14]. On the other hand, exploration of Pb contamination in the same supplement collection showed a strong relationship with country of origin: products made in India and the EU were linked to an increased risk when compared with products made in the USA, Canada, and Australia. The overall conclusion was that supplement formulation, dosage form, and country of origin were strong determinants of elemental contamination that may be attributable to the high number of ingredients or the containing herbs [14]. A Polish study investigating supplements from several continents confirmed the differentiation of elemental contamination and the country where the supplement was produced, whereas dosage form and the type of the principal ingredient exerted lesser influence [15]. Another study found that the risk of reaching high intakes of toxic elements was strongly associated with marine ingredients (e.g., algae) and to a lesser extent with terrestrial plant-based products [16]. The same study highlighted the extremely wide ranges of concentrations of certain elements (including Pb, Cd, and As), varying by several orders of magnitude more than in most other commercial food categories [16]. On the other hand, medicinal plant supplements from the Polish market exhibited similar frequency of occurrence of at least one of the analyzed elements (Pb, Cd, and Hg) regardless of their terrestrial plant or microalgae origin (79.2 and 88.2%, respectively), while mean concentrations of Pb and Cd were substantially higher in the former than the latter (Pb 0.419 ± 0.896 vs. 0.131 ± 0.130 mg/kg; Cd 0.077 ± 0.191 vs. 0.027 ± 0.026) [15].

The European Union (EU) Regulation 915/2023, which has been transposed into Serbian legislation, has established maximum allowed concentrations for only three elements in food supplements (3.0, 1.0, and 0.10 mg/kg for Pb, Cd, and Hg, respectively) [17]. National pharmacopoeias and international guidelines provide the maximum allowed amounts of elements in pharmaceutical products. The International Council for Harmonization of Technical Requirements for Pharmaceuticals for Human Use (ICH) Guideline for Elemental Impurities [18] has established the permitted daily exposure (PDE) to elements that have been placed into three classes based on their toxicity and likelihood of occurrence in a pharmaceutical product. Class 1 elements such as As, Cd, Hg, and Pb, severe environmental contaminants and systemic toxicants causing multiple organ injury and failure even in small doses [19,20,21,22], have limited or no use in the manufacture of pharmaceuticals. Class 2 elements have generally been considered route-dependent human toxicants, and they were further divided into sub-classes 2A and 2B based on their relative likelihood of occurrence in the product: Class 2A (elements with relatively high probability of occurrence): Co, V, and Ni; and Class 2B (elements with reduced probability of occurrence): Au, Ir, Os, Pd, Pt, Rh, Ru, Ag, Se, and Tl. Class 3 elements have relatively low toxicity by the oral route of administration: Ba, Cr, Cu, Li, Mo, Sb, and Sn. The elements for which PDEs have not been established due to their low inherent toxicity are Al, B, Ca, Fe, K, Mg, Mn, Na, W, and Zn. A need for simple and reliable methods for determining the element content of products available on the market has to be emphasized.

According to the analysis of trends on the European food supplement market, a substantial increase in the market value from 14.95 billion dollars in 2019 to 33.80 billion by 2027 was anticipated, along with a drastic increase in the use of supplements of natural origin [23]. Serbia has not been exempt from the upward trend in the use of herbal food supplements [24,25]. A national database of food supplements has been established and maintained by the Ministry of Health. The scarcity of the data related to the elemental profiles of the ample supply of herbal food supplements and the absence of quantitative risk assessment were the issues targeted to be resolved with the current study, directed towards the analysis of a wide spectrum of elements in a collection of supplements intended for paediatric and adult consumers seeking beneficial health effects and being mostly unaware of potential adverse health effects associated with supplements’ consumption.

## 2. Materials and Methods

### 2.1. Sample Collection

As the database of food supplements marketed in the Republic of Serbia has not been regularly updated (the last update was in June 2018), ‘field screening’ was necessary to perceive the real market situation. The resulting inventory of the products of interest—herbal food supplements offered at a given moment in pharmacies, drug-stores, and health food stores in the second largest Serbian city, Novi Sad—was the basis for the selection of 52 products representing all the major producers and brands with a significant market share. Samples were taken randomly, in unit packages, as they were available to consumers without a doctor’s prescription. Samples were mostly in the form of syrups or drops, carrying various health claims related to their health effects. All samples were delivered to the accredited laboratory in their original packaging, stored according to the manufacturer’s instructions, and opened right before the analysis, within their usage dates. All relevant data taken from the product’s labels is presented in Supplementary Table S1.

### 2.2. Sample Preparation and Inductively Coupled Plasma Mass Spectrometry (ICP-MS) Analysis

The digestion of 0.5 g of the sample with 3 mL of 67–69% nitric acid (VWR International, Leuven, Belgium) and 0.5 mL of 30% H_2_O_2_ (Merck, Darmstadt, Germany) was carried out in a Teflon digestion tube in a microwave oven (Ethos Easy SK-15 high pressure rotor, Milestone, Brondby, Denmark) for 20 min at 1400 W at 210 °C (p-rate 0.5 bar/s, p 40 bar). Afterwards, the final volume was adjusted up to 25 mL with ultrapure water (resistivity 18 MΩ·cm; GenPure Water Purification System, Thermo Fisher Scientific, Langenselbold, Germany).

Standard solutions (1 g/L) of Ag, Al, B, Ba, Cd, Co, Cr, Cu, Fe, Ni, Sb, Se, Sn, Sr, Te, Tl, and Zn were purchased from CPAchem (Stara Zagora, Bulgaria), As, Be, Mn, Pb, and V from AccuStandard Inc. (New Haven, CT, USA), W from Carl Roth (Karlsruhe, Germany), and Hg from Carlo Erba (Milan, Italy). The mix standard solutions were prepared by mixing and diluting individual stock standard solutions of elements with ultrapure water and nitric acid (67–69%; 0.5 mL in a final volume of 50 mL) in a concentration range of 0.1 to 100 μg/L (with the exception of Hg, 0.01–10 μg/L). The criterion for the evaluation of the calibration curve’s linearity was a coefficient of correlation above 0.998.

Analysis was performed on an ICP-MS spectrometer 7700x (Agilent Technologies, Waldbronn, Germany), equipped with an integrated G3160B autosampler, Ni sample and skimmer cones, a MicroMist nebulizer with PEEK connector, and a quartz spray chamber, under operational conditions previously described [26], presented in Table S2, based on EN ISO 15763 and [7]. Applied analysis mode, quantification (monitored and reported isotopes, corresponding internal standard), and performance verification parameters (limit of quantification, accuracy) are presented in Table S3 and accompanied by proficiency testing results presented in Table S4.

### 2.3. Exposure and Risk Assessment

Exposure assessment was based on elements’ concentrations and doses of supplements recommended for intake by targeted consumers (bearing in mind pharmaceutical forms of supplements, it was considered reasonable to assume that consumers would follow the labeled dosage instructions) [15,16]. Calculations that concerned particular population groups were performed only for those products whose declarations contained instructions for use by a given population group [16]. Thus, a total of 52 samples provided 46 supplements intended for adults (both males and females, over 18 years old), 31 for adolescents (11–18 years), 30 for children (7–10 years), and 29 for preschool children (3–6 years), whereas among the 16 supplements intended for toddlers (1–3 years), 6 marked to be used in line with the paediatrician’s instructions were analyzed but omitted from the exposure calculations, as well as 7 among the 9 supplements recommended for infants (up to 1 year).

Chronic exposure was calculated by multiplying the concentration of each individual element (*i*) present in a food supplement with the recommended daily dose of the supplement [27]:

*EDI_i_* = *C_i_* × *D*/*bw* (1)

where: *EDI_i_* is the estimated daily intake of element *i* (mg/kg bw per day); *C_i_* is the concentration of element *i* (mg/kg) (all non-detected results were replaced with zero); *D* is the maximum recommended daily dose of the supplement (kg per day) (in order to calculate doses in mass units, volume units as given on the product’s label were multiplied by density, measured gravimetrically as a mass of 1 mL of the liquid); *bw* is the body weight (kg). Exposure calculations were based on two scenarios related to the duration of supplement intake: long-term (daily) and short-term (2 weeks).

To characterize the safety/risk for human consumption, the following approaches were used: (1) comparison of the estimated intake of an element through supplement consumption with the permitted daily exposure (PDE); (2) non-carcinogenic risk estimates, given as the hazard quotients (HQ) for individual elements; (3) the hazard indices (HI) as a measure of cumulative chronic risk; (4) the margin of exposure (MOE) approach for either non-carcinogenic or carcinogenic effects; and (5) carcinogenic risk estimates, presented as a lifetime cancer risk (LCR).

1. Element’s contribution to the corresponding oral permitted daily exposure, corrected taking into account average body weight for the age class of consumers, was calculated using the following equation [18]:

% *PDE_i_* = (*EDI_i_*/*PDE_i_*) × 100 (2)

where *PDE_i_* is the permitted daily exposure to element *i* (mg/kg bw per day).

Exposure below 100% of the PDE was considered acceptable.

2. Hazard quotients were calculated as a ratio of chronic exposure to the oral reference dose of an element [28] and expressed as percentages:

*HQ_i_* = *EDI_i_*/*RfD_i_* × 100 (3)

where: *HQ_i_* is the chronic hazard quotient of element *i*; *RfD_i_* is the reference dose of element *i* (mg/kg bw per day).

HQ lower than or equal to 100% corresponds to negligible risk, whereas values above 100% were considered indicators of increased risk [28].

3. The hazard index of a sample was obtained as a sum of the hazard quotients of each element in that sample [28] (in percentages):

*HI* = Σ*HQ_i_* × 100 (4)

where: *HI* is the hazard index.

HI was evaluated using the same criteria defined for HQ.

4. The margin of exposure (MOE) was determined as a ratio of the benchmark dose to the exposure attributable to supplement intake [20,21]:

*MOE_i_* = *BMDL_i_*/*EDI_i_* (5)

where: *MOE_i_* is the margin of exposure (unitless); *BMDL_i_* is the benchmark dose lower confidence interval of element *i* (µg/kg bw per day).

MOE was considered of low health concern if ≥1 [20,21]. It should be noted that non-detected results were substituted with the LOQ (upper bound approach; applied to Pb and As) in order to enable the calculation of MOE values for all of the samples, which could be justified by the fact that elements are ubiquitous contaminants, meaning that there is a reasonable possibility of their presence. However, as such an approach could lead to the overestimation of risk and the analytical method enabled low LOQ levels, it was not applied in calculations of other risk indicators.

5. The determination of lifetime cancer risk was based on the oral slope factor [28]:

*LCR_i_* = *EDI_i_* × *q_i_* (6)

where: *LCR_i_* is the lifetime cancer risk (unitless); *q_i_* is the oral slope factor of an element *i* (kg bw per day/mg) (an estimate of the increased cancer risk from a lifetime oral exposure to a dose of 1 mg/kg bw per day).

LCR was evaluated in respect of the maximum tolerable risk of 1 extra lifetime cancer case per 100,000 persons (the WHO recommended guideline) [29].

Toxicological thresholds, used as a measure of the elements’ inherent toxicity specific for the oral route of exposure, were obtained from the following sources: PDE (presented within Table S5) from the ICH [18], RfD (presented within Table S6), and the oral slope factor for As (1.5 kg bw per day/mg) from the US EPA [30]; BMDL from the EFSA: for inorganic As (0.3–8) µg/kg bw per day [20], for Pb 0.50, 0.63, and 1.5 µg/kg bw per day, for neuro-, nephro- and cardio-toxicity, respectively [21]. The mean body weight of specific population groups in Serbia was taken from the national investigation of the population’s health status (adults 76.3 kg, adolescents 51.8 kg, children 33.2 kg, preschool children 23.1 kg, toddlers 11.9 kg, infants 5 kg) [31]. Whether the acceptable level of a selected risk indicator could possibly be exceeded was checked on a case-by-case basis. The number of products causing unacceptable exposure in each population group, as well as the mean and high levels of exposure, were determined. Considering the limited number of samples intended for a population group, the average of the last quartile was used instead of the 95th percentile in order to maintain the statistical robustness of the high-level exposure.

### 2.4. Data Procesing and Statistical Analysis

For the purpose of comparison, the samples were divided into subgroups according to the targeted population: supplements intended for the paediatric population, exclusively for the paediatric population, and exclusively for the adult population. Additionally, products in the form of syrup were compared with other liquid products, domestic with imported, and those containing ‘exotic’ herbs with the usual ones. Comparisons were performed in terms of elements’ occurrence rates (Pearson’s Chi-squared test) and concentrations (Kruskal–Wallis test) (*p* < 0.05). In order to visualize all the information in the data set and assess the similarities and discrepancies of the elemental composition among the examined herbal food supplements, the principal component analysis (PCA) variable score plot and variable loadings plot were generated. The data were standardized to account for the different magnitudes; thus, the responses and parameters contributed equally to the data set variance and to the principal component calculation. The classification was enhanced using partial least squares discriminant analysis (PLS-DA) to generate variable importance scores for the potential element markers for herbal food supplements. The data were processed with Microsoft Office Excel (v2019) and Statistica v12.5 (Statsoft, Tulsa, OK, USA).

## 3. Results

### 3.1. Profile of Elements in Herbal Food Supplements and Compliance Assessment

The elemental profiles of 52 liquid herbal food supplements comprising 24 elements analyzed in the current study are presented in Table 1 and Supplementary Figure S1. ICH Class 1 elements As, Hg, Pb, and Cd were detected in 55.8, 38.5, 23.1, and 80.8% of the samples, reaching the highest concentrations at 26.1, 70.5, 82.4, and 5.7 µg/kg, respectively. ICH Class 2A elements Co, V, and Ni were found in 63.5, 44.2, and 86.5% of the samples, in concentrations up to 13.0, 111, and 560 µg/kg, respectively. ICH Class 2B elements Ag and Tl were found in 30.8 and 73.1% of the samples, respectively. Among ICH Class 3 elements, Ba was detected in 78.8%, Cr in 36.5%, Cu in 92.3%, Sb in 36.5%, and Sn in 66.7% of the samples. ICH non-classified elements (elements for which PDEs have not been established) Te, Sr, and Be were found in 28.8, 94.2, and 11.5% of the samples, respectively. Among other elements, Mn and Zn were the ones most frequently found (94.2 and 88.5%), as opposed to W (42.3%).

Most elements displayed rather variable concentrations. The widest concentration spans, expressed as a ratio of P95 and the minimum determined values, were recorded for Mn (ratio 231), Zn (207), and Sr (126), followed by Fe > As(43) > Cu > Ag > Ba > B > Sn > Ni > Al > Cr > Co > W > Cd(11) > V > Pb(10) (ratios between 52 and 10), while other elements exhibited concentration differences within an order of magnitude (including Hg with a ratio of 6). Concentration spans described by Swedish researchers [16] were much larger in the case of As, Cd, and Pb (1220, 240, and 220-fold differences between P95 and P5, respectively), which could be attributed to a large number of supplements containing very versatile ingredients. On the other hand, Hg exhibited only a 12-fold difference [16], which could be considered comparable with the current study. Several other studies investigating supplements of mixed origin corroborated the rule of large concentration differences [15,32,33]. Additionally, the box–whiskers plot of elements’ concentrations obtained in the current study, presented in Figure S1, indicates positively skewed distributions of most elements (easily observed through substantial deviation of means from the medians, oriented towards the upper end of the concentration range), meaning that the high contamination levels were associated with a limited number of investigated supplements.

Statistical analysis revealed significant differences between groups of supplements intended for the paediatric population (“all paediatric” group) and for adults only: with regard to occurrence rates, all six elements causing differences, As, Hg, Ag, Sb, B, and W, were more frequently found in samples targeting only adults; related to concentrations, out of 11 elements showing differences, even 10 were found in higher amounts in samples exclusively for adults—among them class I elements As (*p* = 0.025), Hg (*p* = 0.017), class II Ni (*p* = 0.032), Ag (*p* = 0.0004) and Tl (*p* = 0.008) and class III Sb (*p* = 0.011) (Table 1). It would be reassuring if it could be considered that these results were caused by the producer’s awareness of the special importance of very careful selection of raw materials and production conditions in the case of products intended for the paediatric population.

Further comparisons of supplements based on selected differentiation criteria—products in the form of syrup vs. other liquids; products of domestic vs. imported origin; products made of usual vs. ‘exotic’ herbs—all pointed out the same division, with only a few exceptions. Indeed, the previous division based on targeting populations was a subset of the new one, so the extent of matching results was not surprising. Thus, other liquids/imported products/‘exotic’ herbs, in addition to As, Hg, Ag, Sb, B, and W, showed a higher frequency of occurrence of Ni, Zn, Ba, and Tl, while elements showing higher concentrations, As, Hg, Ni, Ag, Tl, Sb, B, Mn, Sr, and W, were accompanied by Cu, Zn, and Ba. The main compositional difference between syrups and other liquids was the presence of sugars in syrups, but sugars were not considered a likely cause of their lower elemental contamination. However, further examination of the product composition pointed to the herbal ingredients as the most probable cause of the distinction. Namely, although herbal ingredients as a class were a common element of investigated supplements, they were very diverse in the sense of individual representatives. Thus, in contrast to domestic products containing herbs typically grown on the territory of Serbia (south-east Europe), imported ones contained rather ‘exotic’ plants such as Siberian ginseng, guarana, acerola, echinacea, etc., from unknown, probably non-European origin (although all producer companies were based in Europe). Therefore, the analysis pointed to the country of origin of herbal ingredients as the most probable cause of differentiation between supplements analyzed in the current study, as suggested by several previous reports [14,15].

In order to provide a better insight into the similarities and differences between examined samples, principal component analysis (PCA), including data on elemental concentrations, was undertaken. Despite numerous significant differences in relation to elements’ concentrations revealed by the Kruskal–Wallis test, PCA showed that the first two eigenvalues of the correlation matrix account for a modest amount of 24.01 and 14.80% (PC1 and PC2, respectively) of the total variance of the dataset (Figure S2A). The factor loadings of each element are presented in Figure S2B. The majority of samples belonged to the central group, implicating a similar elemental profile and making it difficult to distinguish the contributions of individual variables. The obvious separation of samples 1 and 2 was characterized by a higher content of W, Ag, Hg, and Tl, which, in general, had notable variable importance (Figure S2C). Furthermore, the same trend of separation along the positive PC1 axis was observed regarding some other samples from the group of other liquids/imported/exotic supplements for adults (3–10, 13, 15). The close positioning of other liquids/imported/exotic samples (10 for adults) in the distinguished group of paediatric/usual samples (14, 24, 34, 36) is mostly due to the high Sr, Mn, and Fe content rather than similarity in the overall elemental profile. Propolis samples 35 and 36 were clearly singled out. Anyway, according to the obtained results and shown discriminating power (Figure S2C), elements W, Ag, Cr, Hg, and V may be suggested as candidate differentiation markers for examined samples belonging to the group of imported/exotic *vs.* domestic/usual supplements. A recent investigation of the herbal supplements on the Croatian market also observed a central grouping of the samples, while Se, Na, Fe, Co, Tl, and Zn were the elements established as the potential markers with the greatest discriminating power between tested supplements [34].

With regard to regulatory compliance, the highest recorded concentrations of three elements specified in Regulation 915/2023 were lower than the maximum allowed ones (max Pb 0.082 mg/kg, Cd 0.006 mg/kg, and Hg 0.070 mg/kg), thus verifying the compliance of all investigated samples. Other studies also reported either no violations [16] or that Pb and Cd may occasionally approach or rarely exceed the maximum allowed limits [15]. On the contrary, monitoring of mineral (in combination with vitamins, amino acids, and some other chemicals) supplements produced in the European Union showed Pb concentrations above the maximum allowed level in all but 6.5% of the products where it was detected [32]. It is noteworthy that in some cases, compliance with the maximum allowed content does not guarantee that the intake of toxic elements will not be significant in comparison with the respectable toxicological thresholds and vice versa, and that is why the risk assessment is crucial for decision-making.

Previous research has shown a wide distribution and high concentration variability of elements in herbal-based products, presumably due to the factors discussed in the Introduction section. Therefore, the results of different studies will be greatly affected by the selection of the samples (this applies even for a sample collection uniform in terms of the type of main ingredient and dosage form), and comparison of the findings should be taken with a grain of salt. However, substantial similarities have been noted between the current and several previous studies. In the aforementioned Croatian study, macro elements (Na, K, Ca, and Mg) were found to be the major constituents, while B, Al, Mn, Fe, Zn, and Sr were in the middle concentration range (31.8–1050 mg/kg), and Se, V, Cr, Co, Ni, Cu, As, Mo, Cd, Sb, Hg, Tl, and Pb were present in trace and ultra-trace amounts (21.7–11,300 μg/kg) [34]. An earlier investigation of food, herbal materials, and food supplements from the Croatian market investigated Ag, As, Ba, Cd, Co, Cr, Cu, Mo, Ni, Pb, Sn, and Hg content and found that heavy metal levels were below the internationally established limits [9]. In mint leaves and chamomile flowers collected in pharmacies in Poland and analyzed for the presence of Cd, Cr, Ni, and Pb, the Cd content exceeded the WHO standards [35]. Brodziak-Dopierala et al. [36] studied Hg content in food supplements available on the Polish market and reported that Hg was present in all samples containing one or more herbal ingredients in highly variable amounts (0.02–4293.07 µg/kg), with two samples exceeding the permitted level. These findings are contradictory to the ones obtained in the current study, both in terms of the reported Hg maximum concentration level and range (much higher and wider, respectively). Another Polish study investigated Hg content of a group of drugs and supplements: Hg content varied from 0.9–476.1 ng/g in drugs, with higher levels being present in prescription drugs than in over-the-counter drugs, while supplements showed somewhat lower Hg content (0.9–16.7 ng/g) [37]. All analyzed herbal supplements from the Turkish market showed detectable amounts of Ni, Zn, Mn, Fe, Al, and Cu, but below allowed limits [38]. In a study conducted in Jordan, Pb, Al, and Ni were present in 88, 76, and 4% of the samples of herbal pharmaceutical products intended for infants, as well as in 93, 87, and 13% of traditional herbs, respectively, while Cd and As, contrary to the current and majority of other studies related to herbal supplements [9,14,15,16,34], were not found in any of the samples [12]. Results of a Brazilian study performed on 74 solid food supplements revealed Pb contamination in the range from 0.04 to 7.01 µg/g, with the highest values related to the samples based on herbal extracts [39]. Analysis of selected Asian herbs used in traditional Chinese medicine and several European herbs and supplements based on these herbal raw materials showed significantly different concentrations of toxic elements between the samples, as observed also in the current and some other studies [16], as a consequence of genetic, agronomic, geographical, and environmental differences in the environment and composition of the soil, as well as the extraction processes [40,41]. The need for more adequate monitoring and control of natural products was emphasized by the alarmingly high concentrations of Pb and Hg found in 20 analyzed Chinese, Arabic, Ayurvedic, and other natural products, as 82% of the samples were above the EU limit for Pb and 62% for Hg. Elevated levels of these elements in the blood and clinical signs of intoxication showed evidence of a significant toxicological and public health risk, as well as a recklessly low awareness of the possibility of intoxication with natural products [42]. Costa et al. [43] reviewed the *Rapid Alert System for Food and Feed* (RASFF) notifications from 1998 to 2018. The relevance of elemental contamination (Pb, Hg, As, Zn, Ni, V, Cd, Cu, Cr, Co, and Tl) has been clearly demonstrated by the number of cases reported (117) and proportion of “seriously” risky products (45%). Pb, Hg, and As were particularly worrying in view of their high toxicity and frequent presence in supplements. Pb dominated with more than 40 notifications; Hg followed very closely; and the number of As-contaminated supplements was almost 2-fold lower. Moreover, a serious increase in the number of cases has been reported over the years.

### 3.2. Exposure and Risk Assessment

The fact that most consumers take the same food supplement every day as opposed to most other foodstuffs, for which the concentration differences are more easily evened out by the consumption of different kinds of products from different producers, provides a powerful argument for uncompromising quality control of these products but also has implications from a risk perspective. Wide concentration ranges of elements are converted into large intake spans, which are in most cases associated with no or negligible risk, but in the case of highly contaminated products, the risk could be serious [16]. Considering the aforementioned concentration patterns of elemental contaminants present in supplements investigated in the current study as well as the findings of previous research on the same topic, the risk was assessed for each tested supplement individually and then complemented with mean and high risk estimates reflecting the sample collection in its entirety (limited to the targeted population). The exposure calculations were based on two consumption scenarios: one to support daily use of supplements over a prolonged period of time (long-term exposure) and one to reflect the period expected to be covered by one package of a supplement, i.e., 2 weeks (short-term).

#### 3.2.1. PDE Approach

**Figure 1:**
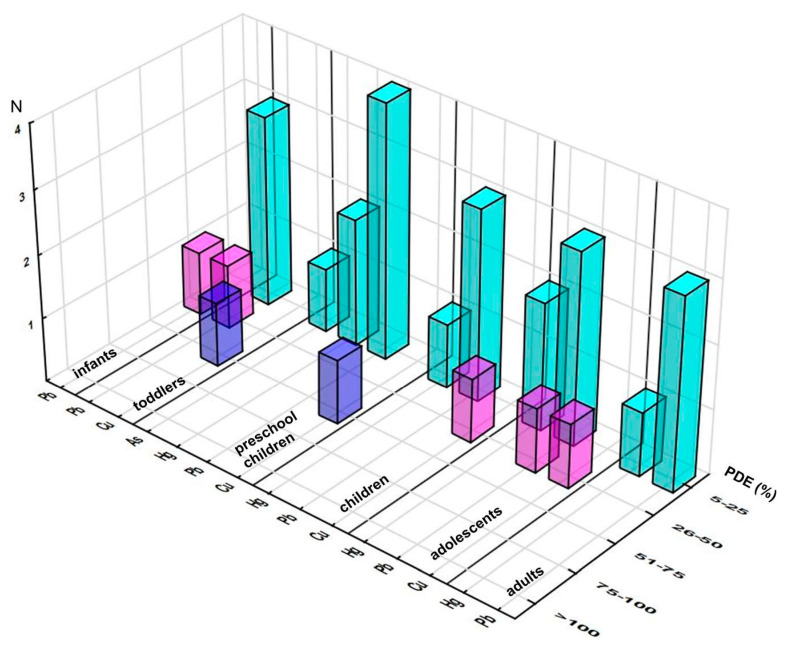
Distribution of the samples according to elements’ contributions (%) to the respective Permitted Daily Exposure (PDE) across the population groups (number of the samples in selected range of %PDE). Note: only elements reaching at least 5% of PDE are presented.

The estimated daily intake of each element through consumption of the analyzed supplements was found to be lower than its corresponding PDE level (Table S5). A daily contribution higher than 5% of PDE was reported for only four elements, three of them from ICH class I (As, Hg, and Pb) and one from class III (Cu). The distribution of elements (across quartiles) clearly showed that the majority of them were enclosed in the lowest interval (5–25%) (regardless of a population group of interest), only Pb and Cu exceeded the thresholds of the second quartile, whereas the third one accommodated only Cu (one sample), and not one of the elements reached the highest one (Figure 1).

The maximum achieved contributions to respective PDEs are presented in Figure S3, emphasizing exposure to Pb as being of interest in each population group and Cu as the element with the highest contribution, e.g., for infants, 46.8% Pb, for toddlers, 67.2% Cu and 39.3% Pb, and for preschool children, 52.0% Cu and 20.3% Pb. These two elements also dominated the mean/high level of PDE contribution: for toddlers, 6.9/34.0% Cu and 8.5/29.4% Pb; for preschool children, 1.9/7.6% Cu and 2.1/8.8% Pb (further decreasing with age) (Table S5). Considering that Pb was a class I element, these levels could not be considered harmless, even though the high levels were substantially lower than the maximum ones. Among other elements, the ones contributing more than 1% of PDE at high exposure levels were class I elements As (up to 2.1%, toddlers), Cd (up to 2.9%, toddlers), and Hg (maximum 3.4%, preschool children); then V and Ni (class II) and Ba (class III) (with the exception of V and Ba in adults), whereas the mean contribution levels remained below 1% of PDE for any supplement-element-consumer combination. Chronic exposure to Cd has been associated with renal disease, thyroid disruption, and weakened bones; Hg might cause neurological disorders, motor, renal, cardiovascular, immune, and reproductive dysfunction [8,11].

#### 3.2.2. HQ Approach

**Figure 2:**
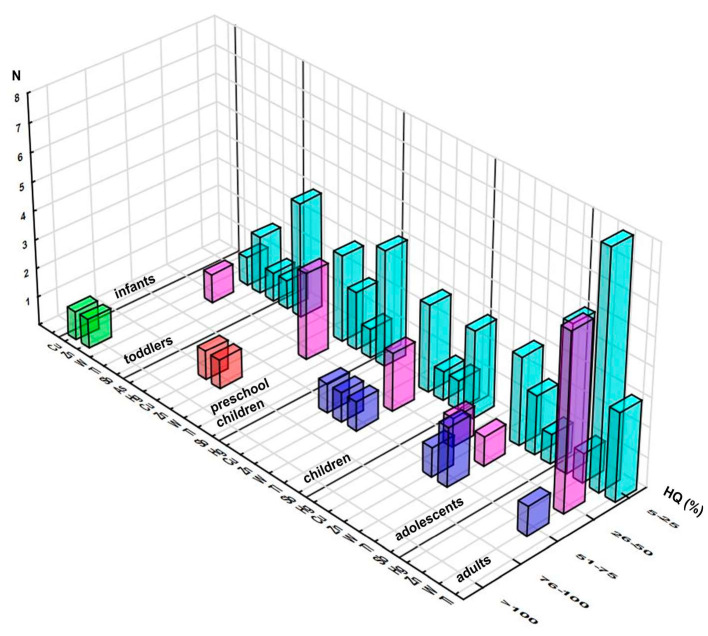
Distribution of the samples according to elements’ contributions (%) to the respective Reference Dose (RfD) across the population groups (number of the samples in selected range of %HQ). Note: only elements reaching at least 5% of respective RfD are presented.

The hazard quotient approach revealed violations of the acceptable level of HQ related to Cu (101%), contrary to the PDE approach, and Zn (113%) in one sample (based on astragalus and Siberian ginseng) when consumed by toddlers (Figure 2, Table S6). Additional five elements exceeded 5% of their respective RfDs: As, Hg, W, Tl, and Sb, as presented in Figure 2, displaying the distribution of the obtained HQs.

#### 3.2.3. HI Approach

**Figure 3:**
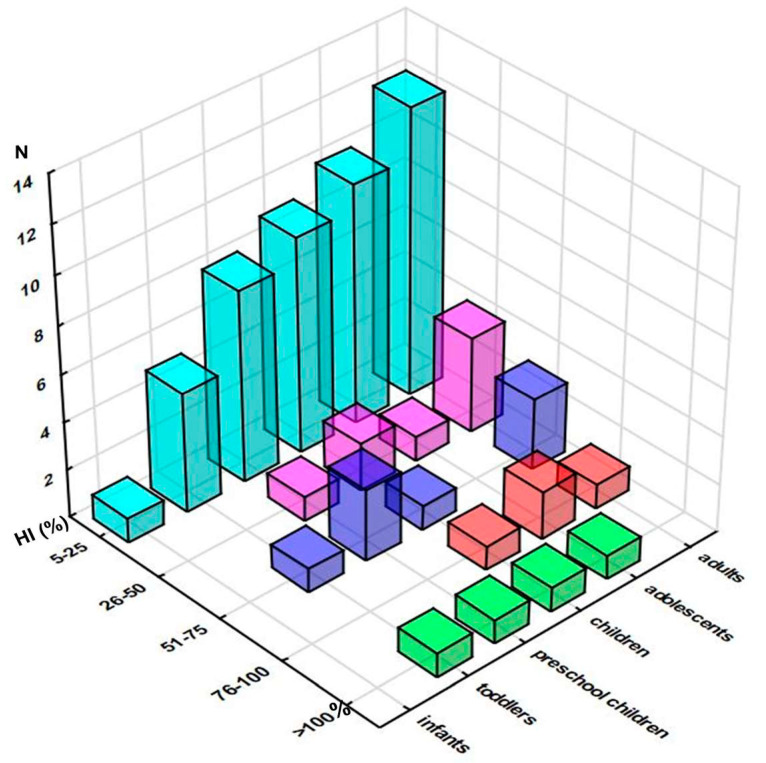
Distribution of the samples according to their Hazard Indices (HI) across the population groups (number of the samples in selected range of %HI). Note: only samples reaching at least 5% of HI are presented.

Hazard index values greater than 100% were obtained for only one of the investigated samples (Table S6), intended for toddlers (229%), preschool children (177%), children (123%), and adolescents (105%) (Figure S5). The distribution of the obtained HI values, presented in Figure 3, as expected, resembled the one of HQ: the vast majority of the samples did not exceed the 25% level, and the following quartiles were populated with up to four samples (adults), three (preschool children and adults), and two samples (adolescents).

High-level HI exceeded 100%, although only in toddlers (145%), while in other paediatric groups it ranged from 49.9 to 63.2% (adolescents and preschool children, respectively). On the other hand, the mean HI level was between 13.8 and 35.3%, ascending from adults to toddlers (Table S6). Figure S6 presents 10 elements with a mean contribution to HI of at least 5%, revealing that for most of the samples, HI has largely been driven by W (up to 28.1% in infants), As (up to 21.8% in infants), and Tl (up to 20.7% in children and adolescents), followed by Cu, Co, Hg, Zn, Fe, Ni, and V. It should be noted that a more realistic exposure scenario, assuming 2-week exposure during a year, resulted in a substantial decrease of both HQ and HI values, showing that the recommended amount of intake of investigated supplements should not be considered a health concern, as maximum HI varied between 3.2 and 8.8%, high HI between 1.6 and 5.6%, ascending from adults to toddlers, while mean HI exceeded 1% only for toddlers (1.4%) (Table S6).

#### 3.2.4. MOE Approach

**Figure 4:**
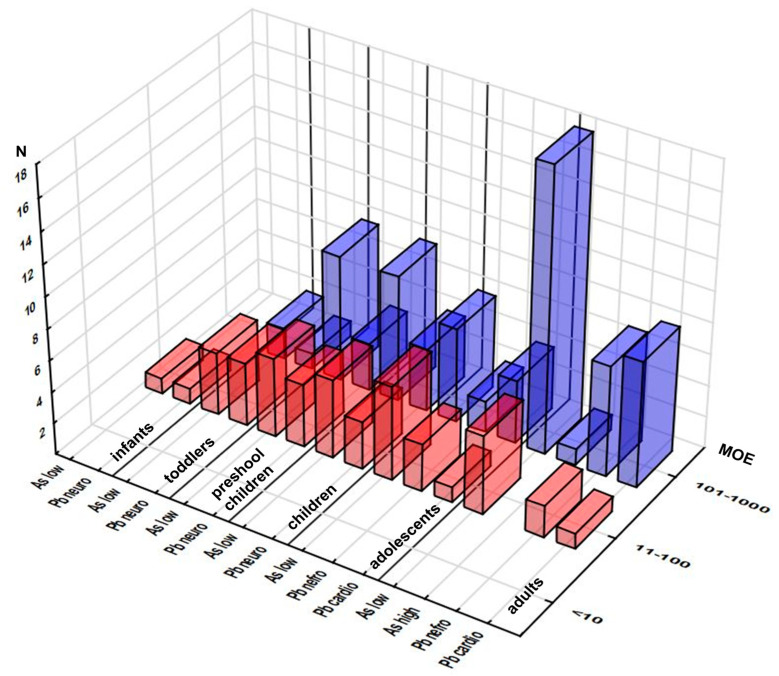
Distribution of the samples according to their Margins of Exposure (MOE) related to As and Pb effects across the population groups (number of the samples in selected MOE range). Note: only samples with MOEs up to 1000 are presented.

The margin exposure approach was used to estimate the risk associated with exposure to As and Pb, elements with established BMDL (Table S7). Exposure to As was below the As BMDL range, and exposure to Pb was below the BMDL regardless of the type of adverse health effect and exposed population group (Figure 4, Table S7). Moreover, not one MOE below 10 was reported. MOE values in the range from 11 to 100 were reported in each population group, both for As considering the BMDL lower limit (infants 1 sample, toddlers 4, preschool children, children and adults 5 each, adolescents 6) and relevant Pb effects (doses relevant for neurotoxicity for infants (1), toddlers (4), and all children (4), doses relevant for nefro- (3 and 2), and cardiotoxicity (1 each) for adolescents and adults).

The number of data points distributed over the 101–1000 MOE range varied considerably, depending on the population group and selected effect, reaching a maximum of 18 samples for adults’ exposure to As with the lower BMDL limit criterion. The minimum MOE levels obtained for each population group—element/effect combination—are presented in Figure S7, displaying neurotoxicity of Pb as the most prominent concern for infants (MOE 11) and toddlers (MOE 13), while As exposure in preschool children caused the highest concern (MOE 15). MOE corresponding to high As exposure ranged from 50 (toddlers) to 74 (children) in paediatric groups, whereas in the case of Pb, high level MOE for neurotoxicity in toddlers and all children ranged from 19 and 45, while MOE for nephro/cardio-toxicity in adolescents and adults were 54/127 and 82/196, respectively (Table S7). Chronic exposure to As has been associated with dermal lesions, carcinogenic effects, impairment of the reproductive system, and atherosclerosis [20]. Pb exposure could affect neurotransmitter release and haemoglobin synthesis, which might result in nervous, haematological, reproductive, renal, and digestive effects [8,11,35]. Pb has been known to induce renal tumors, reduce cognitive development, increase blood pressure, and cause cardiovascular diseases in adults [8]. Children are particularly sensitive to Pb exposure, leading to decreased intelligence quotient, learning disabilities, and impairments of behavior [8,44].

#### 3.2.5. LCR Approach

**Figure 5:**
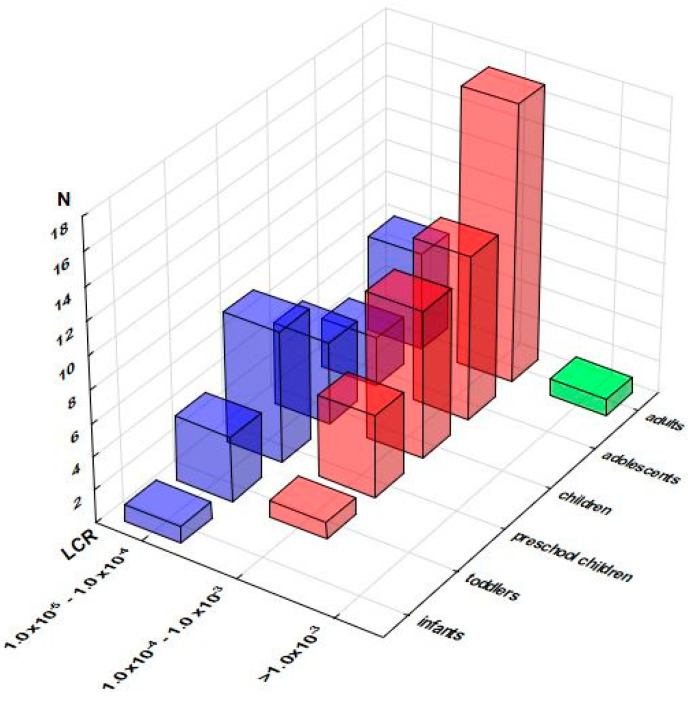
Distribution of the samples according to the consequent Lifetime Cancer Risk (LCR) related to As effects across the population groups (number of the samples in selected LCR range). Note: only samples with LCRs above 1.0 × 10^−5^ are presented.

Estimates of a lifetime cancer risk due to As exposure (Table S7) contrasted HQ and MOE approaches, revealing a considerable number of supplements responsible for the exposure greater than one extra cancer case per 100,000 persons (1.0 × 10^−5^ ), approaching half of the samples intended for preschool children (13 out of 29; 44.8%), children (14 out of 30; 46.7%), and adolescents (13 out of 31; 41.9%), and even exceeding half of the samples in the case of adults (25 out of 46; 54.3%). As presented in Figure 5, for children, adolescents, and adults, a large proportion of these samples surpassed the even level of 1.0 × 10^−4^, with one supplement intended for adults exceeding even 1.0 × 10^−3^.

The highest LCR levels obtained for each population group are presented in Figure S8. In paediatric groups, mean LCR levels ranged between 3.8 × 10^−5^ and 1.3 × 10^−4^, with high LCR levels between 1.1 × 10^−4^ and 4.4 × 10^−4^, lining up from toddlers to adolescents. The adults very closely resembled the adolescents (Table S7). Even a favorable 2-week consumption scenario during a year resulted in 1 sample for preschool children, 2 for children, 6 for adolescents, and 9 for adults surpassing the acceptable risk level, as well as high levels of exposure for children (1.1 × 10^−5^), adolescents (1.7 × 10^−5^), and adults (1.7 × 10^−5^) (Table S7).

Similar to the current study, the content of elemental impurities found in 93 analyzed samples from the Croatian market was below the limits established by the ICH guidelines [10]. The predicted risk of Hg exposure through consumption of food supplements available on the Polish market did not indicate a health threat [37]. The estimated daily intake of Cd, Cr, Ni, and Pb found in mint leaves and chamomile flowers from Polish pharmacies was very low, suggesting that usage of the analyzed herbs could be considered safe [35]. On the contrary, a Romanian investigation of mineral, plant-, and animal-based supplements showed an unacceptable risk of Cd exposure from all types of products, but much greater in the case of animal and mineral ones [45]. In a Jordanian study, 8% of the samples of herbal pharmaceutical products intended for infants exceeded the tolerable daily intake of Pb regulated by the US Food and Drug Administration, while 40, 60, and 8% of the analyzed traditional herbs caused excessive daily exposure to Pb, Al, and Ni, respectively [12]. Based on the USP PDE limits, not one of the 33 supplements used by the Lebanese population posed a health risk due to the intake of Pb, Hg, Cr, Fe, Zn, Mn, Se, and Mo. However, 18, 30, and 62% of the samples were linked to excessive exposure to Cu, Cd, and As, respectively [33]. Not one of the hundreds of diverse supplements available in the United Arab Emirates markets approached the toxicological thresholds intake level of Pb, Cd, or As [14]. The predicted daily intake of Cu, Co, Fe, Ni, Pb, and Zn found in various Nigerian supplements, including herbal, exceeded the USP PDE recommendations, which were met by the other 10 analyzed elements [46]. Daily intake of Pb from 74 solid food supplements from the Brazilian market ranged from 0.04 to 25.3 µg/day, with two samples causing exposure above the USP limit (10 µg/day) [39]. Based on the concentrations of 12 elements determined in six herbal supplements from the USA market, their consumption as recommended on labels did not pose a significant risk, as the amounts on a per serving and daily use basis were all below the recommended daily allowance [47]. A great contribution to understanding the trace and major elemental composition and its implications for health risk associated with food supplements containing rather diverse ingredients has to be acknowledged to Swedish researchers, who reported concentrations for a total of 71 and risk estimates for 23 individual elements and a group of 16 rare earth elements. High levels of Al, As, Fe, Mn, and Pb intake by 3–6 year old children exceeded 50% of the respective toxicological thresholds, while in the case of adult women, that was achieved at the maximum reported intake of the same elements with the exception of Pb and additionally Mo, Se, and Zn [16]. The influence of duration of consumption on the resulting risk estimates was highlighted by Polish researchers investigating medicinal plant food supplements. Changes from 2-week intake to 1-, 2-, or 3-month intake led to a notable increase in the number of examined supplements (including terrestrial plant-based products) exceeding the acceptable intake of Pb, Cd, and Hg, evaluated individually and cumulatively using HQ and HI approaches [15]. The current study also showed that consumption of a random product containing a high amount of As for a period as short as 2-weeks could pose a risk concern, while the risk related to other investigated elements in such a scenario appeared negligible. As the findings of the presented studies do not unequivocally corroborate the safety of herbal food supplements, the importance of quality control in the production process, monitoring of the marketed products, and constant investment in efforts to raise consumers’ awareness have to be highlighted.

An issue of pivotal importance is the extent to which supplements increase the usual daily intake of toxic elements through food. For that purpose, data related to the dietary exposure of the European population to Pb, Cd, Hg, and As were extracted from the European Food Safety Authority (EFSA) assessment reports (Table S8) [22,48,49,50] and used for calculations. Based on the mean intake of elements of interest through the investigated supplements, additional intake appears to be small in the case of Pb and Cd (below 1%) and As (up to 3.1%, depending on the population group), whereas Hg intake reaching a 7.7% increase in the adolescent population attracted considerable attention (Table 2).

The increase of usual dietary intake related to high (4th quartile mean) and maximum intake from supplements remained on a relatively low level for specified elements, except in the case of Hg, with the high intake level responsible for the maximum 14.8% increase in the population of adolescents and the maximum intake causing a worrisome increase of 15.5% for preschool children, 18.0% for children, 38.1% for adolescents, and 29.2% for adults. Although intake of elements through analyzed supplements did not exceed their respective BMDLs/TWIs/RfDs, from a health risk perspective, it has to be taken into account that the usual diet could already result in intakes surpassing toxicologically tolerable levels and thus cause health concerns without even taking into account additional intake from food supplements.

The aforementioned Swedish study reported even more drastic additional intakes of toxic elements (Pb, Cd, As (inorganic), Al, Ni) through food supplements, with As as the most extreme example, reaching a 340% increase in the population of adult women and even 1200% in 3–6 year old children in relation to the usual dietary intake [16]. However, these values were related to the marine (non-fish oil) supplements, whereas products based on terrestrial plants showed results comparable with the current study in the case of adults (median/high/max additional intake for women 0.2/2.2/16%) but substantially higher for children (1.2/31/60%). Regarding Pb and Cd intake from terrestrial plant-based products marketed in Sweden, the most important difference in relation to the current study was observed related to maximum and, in the case of children, high intakes, showing substantially higher levels (Pb max 16 and 57%, Cd max 36 and 130%, for women and children, respectively) [16]. The overall conclusion was that extremely variable and hardly predictable concentrations of toxic elements in food supplements could randomly result in either marginal or significant additions to the usual dietary intake, the latter being especially challenging in the case of sensitive individuals who are more prone to supplement consumption and those using more products simultaneously.

### 3.3. Overall Considerations and Study Limitations

Bearing in mind that an element’s species could have a substantial impact on its toxicological potential, the limited analytical capacity of the current study to produce only a total quantity of such elements has to be acknowledged. Regarding As and Hg, the total amount found in a matrix of herbal preparations could be considered a good surrogate for their inorganic forms, providing adequate toxicological representation of As (organic forms have mainly been considered of no toxicological importance) and inorganic Hg (organometallic forms have more prominent adverse effects but no significant likelihood of occurrence in investigated products) [20,22]. Similarly, Cr in its hexavalent form has been classified as carcinogenic, while trivalent ions have been considered essential trace elements [9]. The precautionary principle could impose that total Cr be used as a surrogate of its haxavalent form, but even that was stultified by the miniscule exposure to Cr through food supplements investigated in the current study.

An additional limitation was the relatively low number of supplements intended for toddlers that have been included in calculations (10), because no dosage instructions were available on the additional 7 products (to be used according to a ‘paediatrician’s instructions’). Thus, estimates of risk should be interpreted with caution. The stipulation of a personal paediatrician’s dosage instructions, as stated on the labels of 7 out of 9 supplements intended for infants, should be considered beneficial from the point of view of general well-being, but the product still has to be proven safe. On the other hand, although chronic exposure over the year probably by far overestimates the real exposure, reducing the exposure period to 2-weeks substantially underestimates the exposure of numerous consumers with unlimited trust in the power of natural strengthening of the organism by supplementation, frequently taking multiple supplements simultaneously over prolonged time periods. Furthermore, it should be noted that Internet supplement retailers were not represented in the current study, despite their expected supremacy in future channeling of the products towards consumers.

Although in the current study pregnant and lactating women were considered only as part of the general adult population, the immense impact of prenatal and early-life exposure on health during the life course and the ability of many toxicants, including several elements, to cross the placental barrier or to be excreted in milk urge very careful consideration in an exposure study. The fact that this group was not singled out from the general adult population needs to be noted as a constraint of the current study. Pb has been associated with a greater risk of premature delivery, reduced postnatal growth, effects on the neurodevelopment of children, and a possible contribution to schizophrenia and dementia in adulthood. Additionally, some types of congenital heart disease, higher blood pressure, and kidney effects may be related to maternal Pb exposure. Cd has been associated with preterm low birth weight, adverse effects on neurodevelopment, worsening of children’s cognitive functioning in preschool, and also with atopic dermatitis. High Hg exposure could be very harmful to fetal and child development, especially to the neurological system [8]. Ni has been linked to facial, skeletal, and eye malformations, and Tl has been linked to low birth weight and adverse effects on the developing autonomic nervous system [44]. Furthermore, the rate of elemental absorption in children is greater than in adults, and the exposure of infants has been associated with more severe adverse effects compared to adults, particularly on their developing central nervous system, resulting in defects in their cognitive abilities [12]. However, as no specific toxicological thresholds have yet been established for infants and young children, the current study was limited to the general usage of the limits established for adults.

It has to be emphasized that most elements belong to ubiquitously distributed contaminants, i.e., they could be found in countless food products, contributing to their aggregate exposure. Additionally, other toxic chemicals, as demonstrated in relation to harmful volatile compounds [51,52] and mycotoxins [53], could pose hidden health risks for consumers of plant-based supplements. An important observation related to the content of supplement labels was the absence of information/warning of the recommended/maximum duration of usage, as observed in other studies as well [15], despite the fact that duration of exposure is one of the main determinants of chronic health risk. Assessment of the risk associated with concurrent exposure to an array of harmful chemicals should be prioritized, especially in cases of substantial exposure in the paediatric population.

Detailed exploration of factors influencing the level of elemental contamination (e.g., ingredients that could be efficient metal accumulators) would require diversification of the sample formulations and dosage forms, an extended list of herbal ingredients to include new species, and other characteristics of interest. Therefore, future research directions should lead to the identification of potential ingredients that could be characterized by higher levels of particular contaminants in order to highlight plausible scenarios of safety improvement in the development of food supplements, as well as the assessment of aggregate and cumulative risks caused by supplement consumption.

## 4. Conclusions

The increased popularity of herbal products and the frequent combination of different herbal food supplements, nowadays mostly considered an element of a healthy lifestyle, highlight the need for assessment of the risk associated with their regular consumption, keeping in mind the health safety of all consumer groups, especially paediatric ones. Risk management decisions based on relevant risk assessment data and directed towards mitigation of early-life exposure to harmful chemicals are pivotal for protecting children’s health. In some circumstances, the use of supplements could be advisable, but leisurely consumption and unjustifiable/excessive usage could increase the likelihood of detrimental health effects related not only to additional exposure to toxic compounds but also to the potential of known or unknown supplement ingredients for interaction with concomitantly used pharmacological products.

Considering widespread public trust in natural/herbal products efficacy and inherent safety, aggressive health-maintaining/improving promotional marketing, the absence of a warning of the recommended usage duration, frequent simultaneous use of multiple supplements, the immense spectrum of versatile potential contaminants, and the huge variability of their amounts and possible interactions, an exhaustive risk assessment accounting for not only supplements in isolation but also their addition to the usual dietary intake seems like an appropriate prerequisite for the market release of these products. Findings from the current and other published research underline the need for stringent controls based on a comprehensive legislative framework that has yet to be developed.

## Figures and Tables

**Table 1:** Summary of elemental profiles of herbal food supplements.

Element	All (52)			All Paediatric (35)			Paediatric Only (6)			Adults Only (17)		
	N (%) Detected	Mean ± SD (µg/kg)	Max (µg/kg)	N (%) Detected	Mean ± SD (µg/kg)	Max (µg/kg)	N (%) Detected	Mean ± SD (µg/kg)	Max (µg/kg)	N (%) Detected	Mean ± SD (µg/kg)	Max (µg/kg)
Be	6 (11.5)	0.3 ± 0.8	3.5	3 (8.6)	0.2 ± 0.7	2.9	0 (0)	0.0	0.0	3 (17.6)	0.4 ± 0.9	3.5
B ^a,b^	32 (61.5)	826 ± 2118	14,685	15 (42.9)	275 ± 594	3050	5 (83.3)	464 ± 468	1327	17 (100)	1961 ± 3394	14,685
Al	27 (51.9)	555 ± 834	3515	15 (42.9)	430 ± 704	2641	2 (33.3)	495 ± 1060	2642	12 (70.6)	813 ± 1030	3515
V	23 (44.2)	12.7 ± 21.5	111	16 (47.1)	12.7 ± 18.3	79.2	3 (50.0)	23.4 ± 32.7	79.2	7 (41.2)	13.4 ± 27.9	111
Cr	19 (36.5)	41.1 ± 86.8	454	12 (35.3)	36.9 ± 73.1	358	3 (50.0)	97.3 ± 147	358	7 (41.2)	52.1 ± 113	454
Mn ^b^	49 (94.2)	1132 ± 3277	20,846	32 (91.4)	359 ± 717	3717	6 (100)	577 ± 786	2130	17(100)	2725 ± 5397	20,846
Fe	31 (59.6)	2564 ± 6012	38,400	19 (54.3)	1723 ± 2849	11,879	4 (66.7)	2661 ± 3276	8255	12 (70.6)	4297 ± 9656	38,400
Co	33 (63.5)	1.5 ± 2.2	13.0	21 (60.0)	1.5 ± 2.5	13.0	5 (83.3)	4.0 ± 4.6	13.0	12 (70.6)	1.5 ± 1.5	4.2
Ni ^b^	45 (86.5)	57.0 ± 91.7	560	28 (80.0)	45.8 ± 92.7	560	6 (100)	140 ± 207	560	17 (100)	80.1 ± 87.6	385
Cu	48 (92.3)	917 ± 5774	41,745	32 (91.4)	1279 ± 7041	41,745	6 (100)	7086 ± 16,978	41,745	16 (94.1)	172 ± 147	473
Zn ^b^	46 (88.5)	10,531 ± 50,532	349,961	29 (82.9)	11,471 ± 59,001	349,961	6(100)	61,787 ± 141,336	349,961	17 (100)	8598 ± 27,131	11,365
As ^a,b^	29 (55.8)	3.9 ± 6.0	26.1	16 (45.7)	2.2 ± 3.8	16.7	5 (83.3)	4.8 ± 6.7	16.7	13 (76.5)	7.2 ± 8.1	26.1
Sr ^b^	49 (94.2)	316 ± 468	1955	33 (94.3)	178 ± 336	1760	5 (83.3)	393 ± 674	1760	16 (94.1)	599 ± 577	1955
Ag ^a,b^	16 (30.8)	17.8 ± 43.3	238	4 (11.4)	1.7 ± 6.1	33.5	0 (0)	0.0	0.0	12 (70.6)	51.1 ± 64.5	238
Cd	42 (80.8)	1.2 ± 1.1	5.7	27 (77.1)	1.1 ± 1.2	5.7	6 (100)	2.4 ± 2.0	5.7	15 (88.2)	1.5 ± 1.0	3.0
Sn	34 (66.7)	15.5 ± 19.7	83.0	24 (70.6)	19.2 ± 21.6	83.0	3 (50.0)	22.0 ± 28.7	63.6	10 (58.8)	8.1 ± 12.7	51.7
Sb ^a,b^	19 (36.5)	1.6 ± 3.3	20.0	7 (20.0)	1.1 ± 3.6	20.0	3 (50.0)	1.0 ± 1.1	2.2	12 (70.6)	2.5 ± 2.5	7.8
Te	15 (28.8)	1.2 ± 2.3	10.0	8 (22.9)	1.0 ± 2.4	10.0	2 (33.3)	2.0 ± 3.5	8.7	7 (41.2)	1.6 ± 2.3	7.3
Ba	41 (78.8)	354 ± 662	4436	25 (71.4)	351 ± 792	4436	6 (100)	1238 ± 1700	4436	16 (94.1)	360 ± 254	904
W ^a,b^	22 (42.3)	196 ± 328	1512	9 (25.7)	52.0 ± 106	400	4 (66.7)	78.0 ± 62.2	146	13 (76.5)	491 ± 426	1512
Hg ^a,b^	20 (38.5)	11.2 ± 17.5	70.5	8 (22.9)	6.4 ± 13.3	55.0	1 (16.7)	3.6 ± 8.8	21.7	12 (70.6)	21.1 ± 21.2	70.5
Tl ^b^	38 (73.1)	0.5 ± 1.1	7.9	23 (65.7)	0.2 ± 0.2	1.1	6 (100)	0.2 ± 0.1	0.4	15 (88.2)	1.0 ± 1.8	7.9
Pb	12 (23.1)	5.2 ± 14.8	82.4	7 (20.0)	4.1 ± 11.7	62.4	2 (33.3)	12.0 ± 25.0	62.4	5 (29.4)	7.3 ± 19.9	82.4

Be—berillyum, B—boron, Al—aluminium, V—vanadium, Cr—chromium, Mn—manganese, Fe—iron, Co—cobalt, Ni—nickel, Cu—copper, Zn—zink, As—arsenic, Sr—strontium, Ag—silver, Cd—cadmium, Sn—tin, Sb—antimony, Te—tellurium, Ba—barium, W—tungsteen, Hg—mercury, Tl—thallium, Pb—lead. N—number of the samples, SD—standard deviation. ^a^—elements with statistically different occurrence rates in “all paediatric” and “adults only” groups (*p* < 0.05); ^b^—elements with statistically different mean concentrations in “all paediatric” and “adults only” groups (*p* < 0.05).

**Table 2:** Percental increase of usual dietary intake of Pb, Cd, Hg (inorganic), and As (inorganic) associated with daily consumption of supplements, based on the mean, high, and maximum intakes derived from the herbal food supplements investigated in the current study.

		Infants	Toddlers	Preschool Children	Children	Adolescents	Adults
Pb	mean ^a^	0.00	0.65	0.22	0.19	0.36	0.22
	high ^c^		1.7	0.56	0.49	0.79	0.51
	maximum ^e^	2.6	2.3	1.3	0.91	2.8	2.2
Cd	mean ^a^	0.00	0.12	0.13	0.12	0.20	0.19
	high ^c^		0.32	0.20	0.19	0.26	0.26
	maximum ^e^	0.04	0.48	0.26	0.36	0.43	0.39
Hg	mean ^a^	0.00	0.88	4.1	3.7	7.7	7.4
	high ^c^		2.8	8.8	8.2	14.8	13.6
	maximum ^e^	0.00	5.1	15.5	18.0	38.1	29.2
As	mean ^b^	0.04	1.3	1.7	1.5	2.7	3.1
	high ^d^		1.7	2.5	2.2	4.0	4.0
	maximum ^f^	1.1	1.0	3.8	2.6	3.2	4.8

The ratios were calculated as: ^a^ mean intake/middle bound (MB) median mean intake [22,48,49]; ^b^ mean intake/lower bound (LB) median mean intake [50]; ^c^ high (4th quartile mean) intake/MB median P95 intake [22,48,49]; ^d^ high (4th quartile mean) intake/LB median P95 intake [50]; ^e^ max intake/MB median P95 intake [22,48,49]; ^f^ max intake/upper bound (UB) median P95 intake [50].

## Data Availability

The datasets generated for this study are available on request to the corresponding author.

## Supplementary Materials

The following supporting information can be downloaded at: https://www.mdpi.com/article/10.3390/foods12142746/s1. Figure S1: Box-Whisker plot of concentrations of elements in herbal food supplements (whiskers extending from min to max, ☐ interquartile range, median, Δ mean); Figure S2: (A) PCA score plot; (B) PCA loadings of variables; (C) Variable importance plot of the candidate element markers for herbal food supplements; Figure S3: Maximum achieved %PDE contributions across the population groups; Figure S4: Maximum achieved %RfD contributions across the population groups; Figure S5: Maximum achieved %HI levels across the population groups; Figure S6: Mean HQ contribution (%) to HI across the population groups. Note: only elements reaching at least 5% of HI are presented; Figure S7: Minimum achieved As and Pb MOE levels across the population groups; Figure S8: Maximum achieved As LCR levels across the population groups; Table S1: Food supplement information; Table S2: Instrument operating conditions for ICP-MS; Table S3: Quantification and performance verification parameters for ICP-MS determinations; Table S4: Proficiency testing results (ICP-MS); Table S5: Risk associated with herbal food supplements—summary of the PDE approach; Table S6: Risk associated with herbal food supplements—summary of the HQ (RfD) approach; Table S7: Risk associated with herbal food supplements—summary of the MOE and LCR approaches. Table S8: Dietary intake of Pb, Cd, Hg (inorganic) and As (inorganic) in μg/kg bw/day in European population.
