# Supplementary material for: Safety Assessment of Herbal Food Supplements: Elemental Profiling and Associated Risk
Source: Foods. 2023 Jul 19;12(14):2746. doi: 10.3390/foods12142746 (PMC10379194; doi:10.3390/foods12142746)
Supplement: Supplementary file 1 [file foods-12-02746-s001.zip › foods-2493452-supplementary.pdf]

Supplementary material

Safety assessment of herbal food supplements: Elemental profiling and associated risk

Table legend		Page
**Table S1**	Food supplement information	2
**Table S2**	Instrument operating conditions for ICP-MS	8
**Table S3**	Quantification and performance verification parameters for ICP-MS determinations	9
**Table S4**	Proficiency testing results (ICP-MS)	10
**Table S5**	Risk associated to herbal food supplements - summary of PDE approach	11
**Table S6**	Risk associated to herbal food supplements - summary of HQ (RfD) approach	12
**Table S7**	Risk associated to herbal food supplements - summary of MOE and LCR approaches	13
**Table S8**	Dietary intake of Pb, Cd, Hg (inorganic) and As (inorganic) in μg/kg bw/day in European population	14

Figure legend		Page
**Figure S1**	Box-Whisker plot of concentrations of elements in herbal food supplements (whiskers extending from min to max, □ interquartile range, − median, Δ mean)	15
**Figure S2.**	A) PCA score plot; B) PCA loadings of variables; C) Variable importance plot of the candidate element markers for herbal food supplements	16
**Figure S3**	Maximum achieved %PDE contributions across the population groups	17
**Figure S4**	Maximum achieved %RfD contributions across the population groups	17
**Figure S5**	Maximum achieved %HI levels across the population groups	18
**Figure S6**	Mean HQ contribution (%) to HI across the population groups. Note: only elements reaching at least 5% of HI are presented	18
**Figure S7**	Minimum achieved As and Pb MOE levels across the population groups	19
**Figure S8**	Maximum achieved As LCR levels across the population groups	19

Table S1. Food supplement information

Code	Sample name	Manufacturer	Ingredients	Purpose	Package	Population group	Dosage	Intake	Max. intake
								mL/day	mL/day
1	**Radis noir**	SuperDiet, France	black radish		20*15mL ampoules	adults	1*15mL	15	15
2	**Cellimine**	SuperDiet, France	green tea, velvet grass, birch, red grape vine, apple flower and apple juice, lemon	normalisation of body mass and body shaping	20*15mL ampoules	adults	1 to 2*15mL	15 to 30	30
3	**Seve imperiale**	SuperDiet, France	black locust honey, sea buckthorn, silver pine, eucalyptus, pollen, propolis	immune system defense, respiratory system relief	20*15mL ampoules	adults	2*15mL	30	30
4	**Drainaflore**	SuperDiet, France	15 plants	detoxification	20*15mL ampoules	adults	1*15mL	15	15
5	**Harpagophytum**	SuperDiet, France	devil's claw	normal functioning of joints	20*15mL ampoules	adults	1*15mL	15	15
6	**Action tonic**	SuperDiet, France	Siberian ginseng, guarana, acerola	fatigue	20*15mL ampoules	adults	2*15mL	30	30
7	**BioSerine**	SuperDiet, France	chamomile, St. John's worth, lavender...	relaxation	20*15mL ampoules	adults	1*15mL	15	15
8	**Ginseng protect**	SuperDiet, France	echinacea, Siberian ginseng, grapefruit	tone and immune system	20*15mL ampoules	adults	1*15mL	15	15
9	**Defenses naturales**	Herbesan, France	echinacea, shiitake, ginseng, zinc	normal function of immune system	20*15mL ampoules	adults	1*15mL	15	15
10	**Tonus et Energie**	Herbesan, France	ginseng, royal jelly, acerola	tone and energy preservation	20*15mL ampoules	adults	1*15mL	15	15
11	**Frutdep immuno**	Erbozeta, San Marino	beta glucan, resveratrol, concentrated fruit juice (apple, pear, orange, mango, pineapple...)	normal function of immune system	20*10mL	adults	1 to 2*10mL	10 to 20	20
						children	1*10mL	10	10
12	**Pepins de Pamplemousse**	SuperDiet, France	grapefruit seed extract		100mL	adults	2*20 drops	1.92g	1.92g
13	**Pepins de Pamplemousse**	SuperDiet, France	grapefruit		50mL	adults	3*10 to 30 drops	2.07-6.21g	6.21g
14	**Nausolvit junior**	Erbozeta, San	ginger	nausea	20mL	children 4 to 10	3*20 drops	3	3

		Marino							
15	**Nausolvit gocce**	Erbozeta, San Marino	ginger, gingerols	nausea	50mL	adults	3*25 drops	3.75	3.75
16	**Expulmo Zmajac**	Esensa, Belgrade, Serbia	primrose and thyme syrup	helps with expectoration	150g	adults and children over 12	3*15mL	45	45
						children 4 to 12	3*5mL	15	15
						children to 4	pediatrician's recommendation		
17	**Expulmo Lunac**	Esensa, Belgrade, Serbia	marshmallow	helps with dry caugh	150g	adults and children over 12	3*15mL	45	45
						children 6 to 12	3*10mL	30	30
						children 3 to 6	3*5mL	15	15
						children to 3	pediatrician's recommendation		
18	**Expulmo Dasa**	Esensa, Belgrade, Serbia	plantago	helps with expectoration	150g	adults and children over 10	3*10mL	30	30
						children 4 to 10	3*5mL	15	15
						children to 4	pediatrician's recommendation		
19	**Pulmint**	Esensa, Belgrade, Serbia	thyme, propolis, ivy	facilitates expectoration	200mL	adults and children over 12	3*15mL	45	45
						children 5 to 12	3*5mL	15	15
						children to 5	pediatrician's recommendation		
20	**Apipulmint**	Esensa, Belgrade, Serbia	primrose, thyme, propolis	in case of expectoration and inflammation of the upper respiratory tract	200mL 150g	adults	3*15mL	45	45
						children over 12	3*5mL	15	15
21	**Risko**	Pharmanova, Obrenovac, Serbia for Hemofarm, Serbia	plantago syrup	normal functioning of the respiratory mucous membrane	125mL	adults and children over 12	4 to 5*10mL	40 to 50	50
						children 5 to 11	4 to 5*10mL	20 to 25	25
						children 3 to 4	2 to 3*5mL	10 to 15	15
22	**Beba**	Pharmanova, Obrenovac, Serbia for Hemofarm, Serbia	marshmallow syrup	normal functioning of the respiratory mucouse membrane	125mL	adults and children over 12	3*5mL	15	15
						children 6 to 12	3*1mL	3	3
						children 3 to 6	4*0.5mL	2	2

23	**Immuno**	Pharmalife, Lecco, Italy	astragalus root and Siberian ginseng root	normal functioning of immune system	200mL	children over 10	2*10mL	20	20
						children 3 to 10	1*15mL	15	15
						children 1 to 3	1*10mL, doctor's recommend.	10	10
24	**Gas Gocce**	Pharmalife, Lecco, Italy	fennel, chamomile, cumin, mint	digestion improvement	30mL	children 1 to 4	15-20 drops	0.54-0.72 g	0.72g
						infants, to 1	5-10 drops, doctor's recommendati on	0.18-0.36 g	0.36g
25	**Experto herb syrup**	Kirka corporation - Kirka Pharma, Belgrade, Serbia	syrup primrose, thyme, honey	helps with expectoration	200mL	adults and children over 12	3*5 do 10mL	15 to 30	30
						children 4 to 12	3*5mL	15	15
26	**Altiprim herbal syrup for throat and respiratory tract**	Josif Pančić, Serbia	marshmallow root, thyme, primrose	facilitates breathing and expectoration	200mL	adults	4*5mL	20	20
27	**Herbifit syrup**	Pharmamed, Travnik, Bosnia and Herzegovina	Iceland lichen syrup	cough relief	150mL	adults	3*15mL	45	45
						small and school children	3*5mL	15	15
						infants	3*2.5mL	7.5	7.5
28	**Mucoplant Trputac solution**	Dr Theiss, Germany	plantago, echinacea	in case of the upper respiratory tract irritation, for normal immune function	100mL	adults	3*15mL	45	45
						school children	3*5mL	15	15
						children 3 to 6	3*2.5mL	7.5	7.5
29	**Bronhoklir**	Pharmanova, Obrenovac, Serbia for Hemofarm, Serbia	thyme, primrose, purple echinacea	maintains health of the respiratory tract of smokers	200mL	adults	3*5mL	15	15
30	**Bronho-san**	Bilje Borča, Borča, Serbia	herbal syrup primrose, thyme, orange mullein	stimulates and facilitates expectoration, mild antiseptic and bactericidal effect, upper respiratory tract	140g	adults and children over 12	3*15mL	45	45
						children 4 to 12	3*5mL	15	15

				inflammation, bronchitis and cold relief					
31	**Broncamil**	Pharmalife, Lecco, Italy	thyme, plantago, immortelle, wild sunflower, pine, eucalyptus	cough syrup	200mL	adults and children over 6	3*15 mL	45	45
						children 3 to 6	2*15 mL	30	30
						children 1 to 3	1*15 mL	15	15
32	**Bronhoklir**	Hemofarm, Serbia	primrose, thyme	cough reduction	200mL	adults and children over 12	3 do 5*5mL	15 to 25	25
33	**Altiprim P**	Josif Pančić, Serbia	marshmallow, primrose, thyme	calming irritated respiratory tract mucous membrane	100mL	children 5 to 10	3*5 to 10mL	15 to 30	30
						children to 5	3*2.5 to 5mL	7.5 to 15	15
34	**Plantago syrup**	Sinefarm, Serbia	plantago, primrose, thyme	beneficial effect on the upper respiratory tract mucous membrane	140g	adults	4*15mL	60	60
						school children	4*10mL	40	40
						preschool children	4*5mL	20	20
35	**Syrup marshmallow with chamomile**	Sinefarm, Serbia	marshmallow, chamomile	calms dry, irritating and reproductive cough	140g	adults	4*15mL	60	60
						school children	4*10mL	40	40
						preschool children	4*5mL	20	20
						children to 3	pediatrician's recommendation		
36	**Syrup primrose with thyme**	Sinefarm, Serbia	primrose, thyme	helps expectoration	140g	adults	4*15mL	60	60
						school children	4*10mL	40	40
						preschool children	4*5mL	20	20
						children to 3	pediatrician's recommendation		
37	**Žak**	Pharmanova, Obrenovac, Serbia for Hemofarm, Serbia	primrose syrup	normal functioning of respiratory mucous membrane	125mL	children over 4	4*5mL	20	20
38	**Bocko**	Pharmanova, Obrenovac, Serbia for	ivy syrup	facilitates expectoration	125mL	adults and children over 12	3*5mL	15	15
						children 6 to 11	2*5mL	10	10

		Hemofarm, Serbia				children 2 to 5	2*2.5mL	5	5
						children to 2	contraindicated		0
39	**Altal syrup**	Esensa, Belgrade, Serbia	marshmallow, common mallow, basil	helps with expectoration and dry cough	150g	adults	3*15mL	45	45
						children over 12	3*5mL	15	15
40	**Mucoplant syrup for good night**	Dr Theiss, Germany	plantago, thyme, chamomile, lemon balm	in case of cough and respiratory tract discomfort	123g 100mL	adults	3*15mL	45	45
						children over 6	3*5mL	15	15
						children to 6	3*2.5mL	7.5	7.5
41	**Syrup Iceland lichen Holyplant**	Jadran galenski laboratorij, Rijeka, Croatia	Iceland lichen	reduces irritation and feeling of dryness, cough and hoarseness	150mL	adults and children over 12	3*5mL	15	15
						children 6 to 12	1 to 2*2.5mL	2.5 to 5	5
42	**Ivy syrup**	TGFarm Medico, Loznica, Serbia	ivy, thyme	helps with dry and productive cough	125mL, 150g	adults	3*5mL	15	15
						adolescents over 12	3*2.5mL	7.5	7.5
						children to 12	not recommended		0
43	**Plantago syrup**	TGFarm Medico, Loznica, Serbia	buckthorn, thyme	helps with dry and productive cough	125mL, 150g	adults	3*5mL	15	15
						children over 4	3*2.5mL	7.5	7.5
						children to 4	not recommended		0
44	**Marshmallow syrup**	TGFarm Medico, Loznica, Serbia	marshmallow, thyme	for healthy upper respiratory tract mucous membrane	125mL, 150g	adults	3*5mL	15	15
						children over 4	3*2.5mL	7.5	7.5
						children to 4	not recommended		0
45	**Beli slez Herbiko natural**	Abela Pharm, Belgrade, Serbia	marshmallow, rosehip	in case of mouth and throat mucous membrane irritation followed by dry cough	125mL	adults and children over 12	3*15mL	45	45
						children 3 to 12	1*5mL	5	5
						children to 3	not recommended		0
46	**Plantago Herbiko natural**	Abela Pharm, Belgrade, Serbia	rosehip, plantago	in case of mouth and throat mucous membrane irritation followed by dry cough	125mL	adults and children over 12	3*15mL	45	45
						children 4 to 12	3*5mL	15	15
						children 3 to 4	3*2.5mL	7.5	7.5

47	**Primrose Herbiko natural**	Abela Pharm, Belgrade, Serbia	rosehip, primrose	as an expectorant and support in the treatment of dry cough	125mL	adults and children over 12	3*15mL	45	45
						children 4 to 12	3*5mL	15	15
						children to 4	not recommended		0
48	**Herboral Syrup with marshmellow and acerola**	GMZ Ervatim, Belgrade, Serbia	marshmallow, chamomile, acerola	in case of mouth and throat mucous membrane irritation followed by dry cough	125mL	adults and children over 12	3*15mL	45	45
						children 4 to 12	3*5mL	15	15
49	**Herboral Syrup with primrose and acerola**	GMZ Ervatim, Belgrade, Serbia	primrose, thyme, acerola	in case of mouth and throat mucous membrane irritation followed by dry cough	125mL	adults and children over 12	3*15mL	45	45
						children 4 to 12	3*5mL	15	15
50	**Propolis with echinacea drops**	Sinefarm, Serbia	propolis, echinacea	strengthening the body's resistance	20mL	adults	3*30 drops		
51	**Propolis for kids drops**	Sinefarm, Serbia	propolis	strengthening the body's resistance	20mL	children 6-12 (for each year +2 drops up to 15 drops)	3*15 drops		
						children to 6	3*11 drops		
						children to 3	3*5 drops		
						children to 1	pediatrician's recommendation		
52	**Propolis drops**	Kovilj Monastery, Serbia	propolis		20mL	adults	3*30 drops		

Table S2. ICP-MS instrument operating conditions.

ICP-MS

Parameter	Analysis mode	
	No Gas mode	He mode
RF- power (W)	1550	1550
Sample depth (mm)	8	8
Nebulizer pump speed (rps)	0.1	0.1
Plazma gas flow (L/min)	15.0	15.0
Carrier gas flow (L/min)	1.2	1.2
Dilution gas flow (L/min)	1.0	1.0
Spray chamber	Water cooled double pass	
Lens voltage	4.5	4.5
Mass resolution	0.8	0.8
Integration time points/ms	3	3
Points per peak	3	3
Replicates	3	3

Notes:

Prior to the analysis, the ICP-MS system was equilibrated for 30 min and then checked in terms of sensitivity, stability and performance, using the diluted tune solution (1 μg/L of each element), whereas auto-tune and calibration mass tests were performed when it was necessary for optimisation of the instrument. Internal standard was added at a constant rate and concentration to all calibration standards and unknown samples.

In order to avoid any cross-contamination and elements contamination from tools used during sample preparation, procedure recommended by the producer of the microwave oven was applied. Briefly, Teflon digestion tubes were carefully washed with water and soap, dried and then kept at temperature of 130-150°C during 3 hours. Cleanliness of digestion tubes was checked by microwave probe, demanding that they remain cool after exposure to microwaves. Volumetric flask made of PFA were cleaned by immersion in 1M nitric acid during 3 days, followed by rinsing with ultrapure water (resistivity 18 MΩ·cm) and drying.*Chemicals:* Nitric acid 65-67% (VWR International, Leuven, Belgium);Tuning solution: cerium (Ce), cobalt (Co), lithium (Li), magnesium (Mg), thallium (Tl), and ittrium (Y) (10 μg/mL each, in 2% v/v HNO_3) (Agilent Technologies, Waldbronn, Germany); Internal standard: the mixture of bismut (Bi), germanium (Ge), indium (In), lithium (Li), lutetium (Lu), rhodium (Rh), scandium (Sc), and terbium (Tb), (10 μg/mL each, in 10% v/v HNO_3) (Agilent Technologies, Waldbronn, Germany).

Instruments: Microwave oven (Ethos Easy SK-15 high pressure rotor, Milestone, Brondby, Denmark), GenPure Water Purification System (Thermo Fisher Scientific, Langenselbold, Germany).

Table S3. ICP-MS quantification and performance verification parameters.

Element	Symbol	Monitored isotopes	Reported isotope	Internal standard (isotope)	Analysis mode	LOQ[a] (µg/kg)	Accuracy[b] (%)
Beryllium	**Be**	9	9	Sc (45)	No gas	1	102.7
Boron	**B**	11	11	Sc (45)	No gas	100	108.9
Aluminium	**Al**	27	27	Sc (45)	He	200	104.6
Vanadium	**V**	51	51	Sc (45)	He	6	101.9
Chromium	**Cr**	50, 52	52	Sc (45)	He	30	99.6
Manganese	**Mn**	55	55	Sc (45)	He	12	102.9
Iron	**Fe**	54, 56	56	Sc (45)	He	300	103.0
Cobalt	**Co**	59	59	Sc (45)	He	0.4	104.5
Nickel	**Ni**	60, 61	60	Sc (45)	He	10	100.1
Copper	**Cu**	63, 65	65	Sc (45)	He	10	96.4
Zink	**Zn**	66, 67, 68	66	Ge (74)	He	80	92.9
Arsenic	**As**	75	75	Ge (74)	He	1.5	101.1
Selenium	**Se**	76, 77, 78	78	Ge (74)	He	40	94.4
Strontium	**Sr**	88	88	Ge (74)	He	6	92.0
Silver	**Ag**	107	107	In (115)	He	5	108.2
Cadmium	**Cd**	111, 114	111	In (115)	He	0.3	93.7
Tin	**Sn**	117, 118	117	In (115)	He	3	-
Antimony	**Sb**	121, 123	121	In (115)	No gas	1.5	103.3
Tellurium	**Te**	125, 126	125	In (115)	No gas	1.5	107.1
Barium	**Ba**	135	135	In (115)	No gas	30	99.4
Tungsteen	**W**	182	182	Bi (209)	No gas	110	-
Mercury	**Hg**	200, 201, 202	average	Bi (209)	No gas	12	105.2
Thallium	**Tl**	203, 205	205	Bi (209)	No gas	0.1	101.7
Lead	**Pb**	206, 207, 208	average	Bi (209)	No gas	8	92.8

[a] Limit of quantification (LOQ) was determined as the concentration corresponding to 10-fold standard deviation for reagent blanks measurements (n = 10) .

[b] Based on the measurements of two standard reference materials (CRMs): 1643f Trace elements in water and 1642d Mercury in water, from the National Institute of Standards and Technology (Gaithersburg, MD, USA).

Table S4. Results of proficiency testing.

PT provider:	IFA		LGC	LGC	LGC	LGC	Fapas	ERA
Year:	2021		2021	2022	2020	2022	2022	2023
Round:	M159A	M159B	AQ599	AQ621	FC290	FC316	07429	Q500
Element	*z-score*[a]							
**Al**	-0.28	0.62	-0.64	-	-	-	-	-0.45
**As**	0.25	0.50	0.0	-1.32	-1.16	0.0	-	-1.72
**Pb**	-0.82	-0.92	-1.18	-0.73	-0.51	0.0	-0.7	-1.37
**Cd**	-0.39	0.15	0.71	-1.44	-	1.00	-0.6	-1.71
**Cr**	0.7	0.72	-0.32	-0.85	-	-	-	-1.63
**Fe**	0.13	1.06	0.84	-	-	-	-0.7	0.07
**Cu**	-0.18	0.15	0.27	-	-	-	0.8	-0.51
**Mn**	-0.32	0.64	-0.59	-	-	-	-	-1.60
**Ni**	0.61	1.13	0.0	-0.68	-	-	-	-1.50
**Zn**	-1.17	-0.54	0.46	-	-	-	-	-0.16
**Se**	-	2.34	0.49	-1.60	-	-	-	0.32
**Hg**	0.38	0.48	0.47	-1.48	-2.02	-	-	-
**Ba**	-	-	-0.13	-	-	-	-	-0.48
**Be**	-	-	-0.14	0.42	-	-	-	-0.62
**Co**	-	-	-0.32	-0.43	-	-	-	-0.24
**Ag**	-	-	0.29	-	-	-	-	-
**Sn**	-	-	-0.2	-1.05	-	-	1.6	-
**Sb**	-	-	-	-1.29	-	-	-	-0.45
**V**	-	-	-0.15	-1.02	-	-	-	-0.13
**B**	-	-	-	-	-	-	-	0.00
**Sr**	-	-	-	-	-	-	-	-1.28
**Tl**	-	-	-	-	-	-	-	-1.96

IFA - Report: IFA Proficiency testing scheme for water analysis. Round 159 Metals, 2021. University of Natural Resources and Life Sciences, Vienna. Department of Agrobiotechnology IFA-Tulln, Tulln Austria.
LGC - LGC Proficiency testing. Water Chemistry Aquacheck, Sample 17C - Metals. Reports for Round 599, 2021; Round 621, 2022. Food Chemistry – Elements in tea matrix, Round FC290, 2020; Food chemistry – Metals in oil, Round FC316, 2022. 1 Chamberhall Business Park, Chamberhall Green, Bury, United Kingdom.
Fapas - Food Analysis Performance Assessment Scheme. Food chemistry proficiency test report: 07429 - Metallic contaminants in grapefruit puree, 2022. The Food and Environment Research Agency, Sand Hutton, York YO41 1LZ, UK.
ERA A Waters Company. Report: WP Trace Metals, Q500, 2023. 16341 Table Mountain Parkway, Golden, Co, USA.
[a]z-score evaluation criterion: scores between -2 and 2 are considered satisfactory.
Notes: Samples LGC FC290 and FC316 as well as Fapas 07429 were food matrices (FC316 and 07429 plant-based) prepared for analysis by microwave digestion procedure applied also for food supplements. All PT samples were analyzed on the ICP-MS instrument operating under the same experimental conditions. Results of proficiency testing do not include W, thus W concentration values presented in the current study should be considered only as indication.

Table S5. Risk associated with herbal food supplements - summary of PDE approach.

Element[a]	PDE		% PDE																
	Class	Value (mg/50 kg bw)	**Infants** (0-1 year)[b]		**Toddlers** (1-3 years)[b]			**Preschool children** (4-6 years)[b]			**Children** (7-10 years)[b]			**Adolescents** (11-18 years)[b]			**Adults** (over 18 years)[b]		
			min	max	mean	high[c]	max	mean	high[c]	max	mean	high[c]	max	mean	high[c]	max	mean	high[c]	max
**As**	I	15	0,02	2,9	0.72	2.1	2.5	0.62	2.2	6.8	0.56	1.9	4.7	0.55	1.9	3.2	0.40	1.3	3.3
**Cd**	I	5	0	0,41	0.88	2.9	4.4	0.73	1.7	2.3	0.69	1.7	3.2	0.67	1.6	2.6	0.48	1.1	1.7
**Hg**	I	30	0	0	0.23	1.2	2.1	0.82	3.4	6.0	0.74	3.1	7.0	0.80	3.1	8.0	0.73	2.5	5.4
**Pb**	I	5	0	46,8	8.5	29.4	39.3	2.1	8.8	20.3	1.8	7.6	14.1	2.0	7.6	27.1	1.1	4.2	18.4
**V**	IIA	100	0	0	0.58	2.6	3.0	0.49	1.6	2.6	0.40	1.3	2.4	0.47	1.6	2.3	0.24	0.83	1.6
**Co**	IIA	50	0,09	0,23	0.16	0.46	0.57	0.09	0.24	0.34	0.08	0.23	0.41	0.08	0.24	0.47	0.06	0.17	0.32
**Ni**	IIA	200	1,0	1,1	0.84	1.9	2.9	0.55	1.3	2.0	0.52	1.3	2.1	0.52	1.1	1.4	0.44	1.0	1.9
**Se[d]**	IIB	150	0	0	0	0	0	0	0	0	0	0	0	0	0	0	0	0	0
**Ag**	IIB	150	0	0	0.02	0.11	0.23	0.04	0.18	0.64	0.04	0.17	0.45	0.04	0.14	0.41	0.18	0.68	2.1
**Tl**	IIB	8	0,004	0.07	0.20	0.59	0.69	0.10	0.28	0.42	0.09	0.27	0.50	0.10	0.30	0.95	0.10	0.30	0.97
**Cr**	III	11000	0	0	0.02	0.09	0.11	0.01	0.04	0.09	0.01	0.04	0.08	0.01	0.03	0.08	0.01	0.02	0.06
**Cu**	III	3000	0,3	0,12	6.9	34.0	67.2	1.9	7.6	52.0	1.3	5.4	36.2	1.1	4.1	30.9	0.08	0.18	0.28
**Sn**	III	6000	0	0,02	0.02	0.06	0.08	0.01	0.04	0.07	0.01	0.04	0.06	0.01	0.04	0.10	0.01	0.02	0.07
**Sb**	III	1200	0	0	0.01	0.07	0.12	0.01	0.03	0.13	0.01	0.03	0.13	0.005	0.02	0.08	0.003	0.01	0.06
**Ba**	III	1400	0,48	2,6	1.2	3.2	3.7	0.55	1.6	2.8	0.53	1.6	3.0	0.48	1.4	3.4	0.33	0.92	2.3

PDE – Permitted Daily Exposure.
[a]elements with established PDE values [18] (excluded elements: Be, B, Al, Mn, Fe, Zn, Sr, Te and W); [b]according to [31]; [c]high – 4th quartile mean; [d]Se was not detected in any of the samples and 'no exposure no risk' approach was taken.

Table S6. Risk associated with herbal food supplements - summary of HQ (RfD) approach.

Element[a]	RfD (mg/kg bw)	% HQ (%RfD)																
		Infants		Toddlers			Preschool children			Children			Adolescents			Adults		
		min	max	mean	high[b]	max	mean	high[b]	max	mean	high[b]	max	mean	high[b]	max	mean	high[b]	max
**Be**	0.003	0	0	0.01	0.06	0.06	0.004	0.02	0.06	0.004	0.02	0.04	0.005	0.02	0.09	0.004	0.01	0.06
**B**	0.2	0	0,05	0.21	0.80	1.1	0.11	0.41	1.11	0.10	0.38	1.2	0.07	0.25	0.75	0.12	0.38	1.4
**Al**	1	0,02	0,19	0.05	0.21	0.26	0.03	0.12	0.26	0.03	0.10	0.28	0.03	0.10	0.18	0.02	0.07	0.14
**V**	0.005	0	0	0.23	1.0	1.2	0.20	0.64	1.0	0.16	0.52	0.94	0.19	0.62	0.93	0.10	0.33	0.63
**Cr**	1.5	0	0	0.003	0.01	0.02	0.002	0.01	0.01	0.001	0.01	0.01	0.001	0.01	0.01	0.001	0.003	0.01
**Mn**	0.14	0,03	0,07	0.19	0.60	0.63	0.14	0.44	1.5	0.13	0.41	1.1	0.12	0.35	0.87	0.25	0.85	3.2
**Fe**	0.7	0,08	0,62	0.17	0.58	0.64	0.21	0.74	1.8	0.19	0.71	2.5	0.20	0.73	1.6	0.13	0.45	1.7
**Co**	0.0003	0,31	0,76	0.55	1.5	1.9	0.29	0.80	1.1	0.27	0.76	1.4	0.27	0.79	1.6	0.19	0.58	1.1
**Ni**	0.02	0,20	0,22	0.17	0.38	0.58	0.11	0.26	0.39	0.10	0.27	0.42	0.10	0.23	0.28	0.09	0.20	0.38
**Cu**	0.04	0,05	0,18	10.3	51.0	100.9	2.9	11.5	77.9	2.0	8.1	54.3	1.7	6.2	46.4	0.12	0.27	0.41
**Zn**	0.3	0,05	0,40	11.4	56.5	112.7	3.3	13.5	87.1	2.3	9.5	60.7	2.0	7.6	51.8	0.37	1.3	7.5
**As**	0.0003	0,02	2,94	0.72	2.1	2.5	0.62	2.2	6.8	0.56	1.9	4.7	0.55	1.9	3.2	0.40	1.3	3.3
**Se**[c]	0.005	0	0	0	0	0	0	0	0	0	0	0	0	0	0	0	0	0
**Sr**	0.6	0,01	0,02	0.03	0.09	0.13	0.02	0.05	0.13	0.02	0.05	0.14	0.02	0.04	0.09	0.02	0.05	0.09
**Ag**	0.005	0	0	0.01	0.07	0.14	0.03	0.11	0.39	0.02	0.10	0.27	0.02	0.08	0.25	0.11	0.41	1.2
**Cd**	0.0005	0	0,08	0.18	0.59	0.89	0.15	0.34	0.47	0.14	0.34	0.64	0.13	0.31	0.52	0.10	0.23	0.35
**Sn**	0.6	0	0,004	0.004	0.01	0.02	0.003	0.01	0.01	0.00	0.01	0.01	0.003	0.01	0.02	0.001	0.004	0.01
**Sb**	0.0004	0	0	0.79	3.9	7.4	0.39	1.6	7.6	0.37	1.6	7.9	0.29	1.11	5.1	0.19	0.70	3.4
**Ba**	0.2	0,07	0,36	0.16	0.45	0.52	0.08	0.23	0.40	0.07	0.22	0.42	0.07	0.20	0.47	0.05	0.13	0.32
**W**	0.0008	0	1,3	6.4	31.3	49.6	6.3	25.9	49.6	5.3	22.6	64.7	6.2	23.9	66.4	8.6	30.9	60.6
**Hg**	0.0003	0	0	0.46	2.3	4.3	1.6	6.8	11.9	1.5	6.3	13.9	1.6	6.2	16.0	1.5	5.1	10.9
**Tl**	0.00001	0,06	1,1	3.2	9.4	11.1	1.6	4.5	6.7	1.5	4.2	7.9	1.7	4.8	15.3	1.7	4.8	15.5
**HI**	-	2,3	6,9	35.3	145	229	18.1	63.2	177	14.7	51.0	123	15.2	49.9	105	13.8	41.4	82.3
**HI 2w**[d]	-	0,09	0,26	1.4	5.6	8.8	0.70	2.4	6.8	0.56	2.0	4.7	0.58	1.9	4.1	0.53	1.6	3.2

HQ – Hazard Quotient; RfD – oral Reference Dose.
[a]elements with established RfD value [30] (excluded elements: Te and Pb); [b]high – 4th quartile mean; [c]Se was not detected in any of the samples and 'no exposure no risk' approach was taken; [d]exposure over 2 weeks per year.

Table S7. Risk associated with herbal food supplements - summary of MOE and LCR approaches.

Element[a]	BMDL (µg/kg bw/d)	Infants		Toddlers			Preschool children			Children			Adolescents			Adults		
		MOE																
		max	min	mean[b]	high[b,c]	min	mean[b]	high[b,c]	min	mean[b]	high[b,c]	min	mean[b]	high[b,c]	min	mean[b]	high[b,c]	min
**As lower[d]**	0.3	5606	34	1240	50	40	418	70	15	473	74	21	299	68	31	565	104	30
**As upper[e]**	8	149501	906	33072	1340	1078	11138	1861	393	12625	1974	564	7977	1824	828	15056	2767	797
**Pb neuro[f]**	0.5	868	11	160	19	13	106	45	25	136	40	35	101	42	18	260	65	27
**Pb nefro[g]**	0.63	1094	13	201	24	16	133	57	31	171	51	45	128	54	23	327	82	34
**Pb cardio[h]**	1.5	2604	32	479	58	38	318	136	74	407	121	106	304	127	55	779	196	81
	**q** (kg bw per day/mg)	LCR																
		min	max	mean	high[c]	max	mean	high[c]	max	mean	high[c]	max	mean	high[c]	max	mean	high[c]	max
**As**	1.5	4.1×10^{-7}	6.6×10^{-5}	3.8×10^{-5}	1.1×10^{-4}	1.3×10^{-4}	6.5×10^{-5}	2.3×10^{-4}	7.1×10^{-4}	8.3×10^{-5}	2.9×10^{-4}	7.1×10^{-4}	1.3×10^{-4}	4.4×10^{-4}	7.5×10^{-4}	1.4×10^{-4}	4.3×10^{-4}	1.1×10^{-3}
**As 2w[i]**		1.5×10^{-8}	2.5×10^{-6}	1.5×10^{-6}	4.3×10^{-6}	5.1×10^{-6}	2.5×10^{-6}	8.8×10^{-6}	2.7×10^{-5}	3.2×10^{-6}	1.1×10^{-5}	2.7×10^{-5}	4.9×10^{-6}	1.7×10^{-5}	2.9×10^{-5}	5.3×10^{-6}	1.7×10^{-5}	4.4×10^{-5}

BMDL – Benchmark Dose Lower confidence interval [20,21]; MOE – Margin of Exposure; q – cancer slope factor [30]; LCR – Lifetime Cancer Risk.
[a]elements with established BMDL and/or q values; [b]unquantified results were substituted with the LOQ; [c]high – 4th quartile mean; [d]lower level of BMDL range; [e]upper level of BMDL range; [f]neurotoxicity; [g]nefrotoxicity; [h]cardiotoxicity; [i]exposure over 2-weeks per year.

Table S8. Dietary intake of Pb, Cd, Hg (inorganic) and As (inorganic) in µg/kg bw/day in European population [22,48-50].

			Infants	Toddlers	Children	Adolescents	Adults
**Pb**	mean (MB)	min	0.83	1	0.73	0.34	0.4
		max	0.91	1.54	1.27	0.79	0.59
		median		1.31	0.96	0.55	0.5
	P95 (MB)	min	1.8	1.4	1.12	0.58	0.65
		max		2.84	2.01	1.36	0.99
		median		1.73	1.55	0.97	0.83
**Cd**	mean (MB)	min	0.37	0.55	0.45	0.22	0.21
		max	0.39	0.97	0.72	0.45	0.33
		median		0.69	0.56	0.33	0.25
	P95 (MB)	min	0.94	0.76	0.65	0.38	0.35
		max		1.47	1.46	1.00	0.69
		median	0.02	0.11	0.08	0.06	0.04
**Hg** (inorganic)	mean (MB)	min	0.02	0.11	0.08	0.06	0.04
		max	0.18	0.19	0.16	0.10	0.08
		median		0.16	0.12	0.06	0.06
	P95 (MB)	min	0.04	0.19	0.16	0.10	0.08
		max	0.28	0.33	0.32	0.26	0.24
		median		0.25	0.23	0.13	0.11
**As** (inorganic)	mean (LB)	min	0.09	0.12	0.07	0.04	0.03
		max	0.22	0.3	0.17	0.11	0.07
		median	0.15	0.17	0.11	0.06	0.04
	mean (UB)	min	0.26	0.34	0.19	0.1	0.08
		max	0.61	0.61	0.37	0.23	0.15
		median	0.42	0.44	0.3	0.16	0.11
	P95 (LB)	min	0.21	0.24	0.17	0.1	0.07
		max	0.52	0.58	0.37	0.26	0.19
		median	0.36	0.37	0.26	0.14	0.1
	P95 (UB)	min	0.76	0.62	0.41	0.21	0.16
		max	1.2	0.99	0.67	0.44	0.33
		median	0.84	0.75	0.54	0.3	0.21

MB- middle bound, LB – lower bound, UB – upper bound, P95 – 95th percentile.

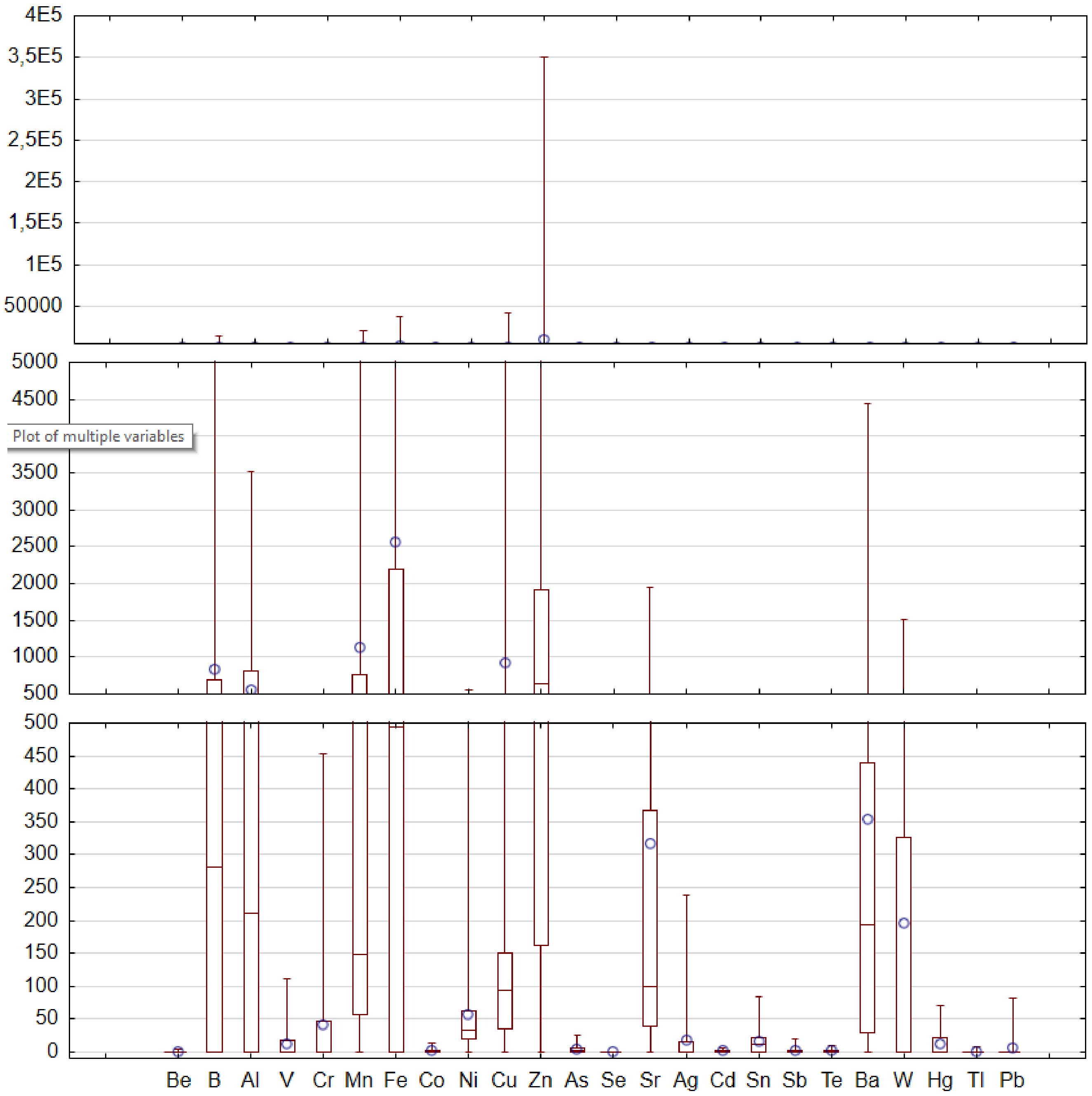

Figure S1. Box-Whisker plot of concentrations (µg/kg) of elements in herbal food supplements (whiskers extending from min to max, □ interquartile range, – median, o mean).

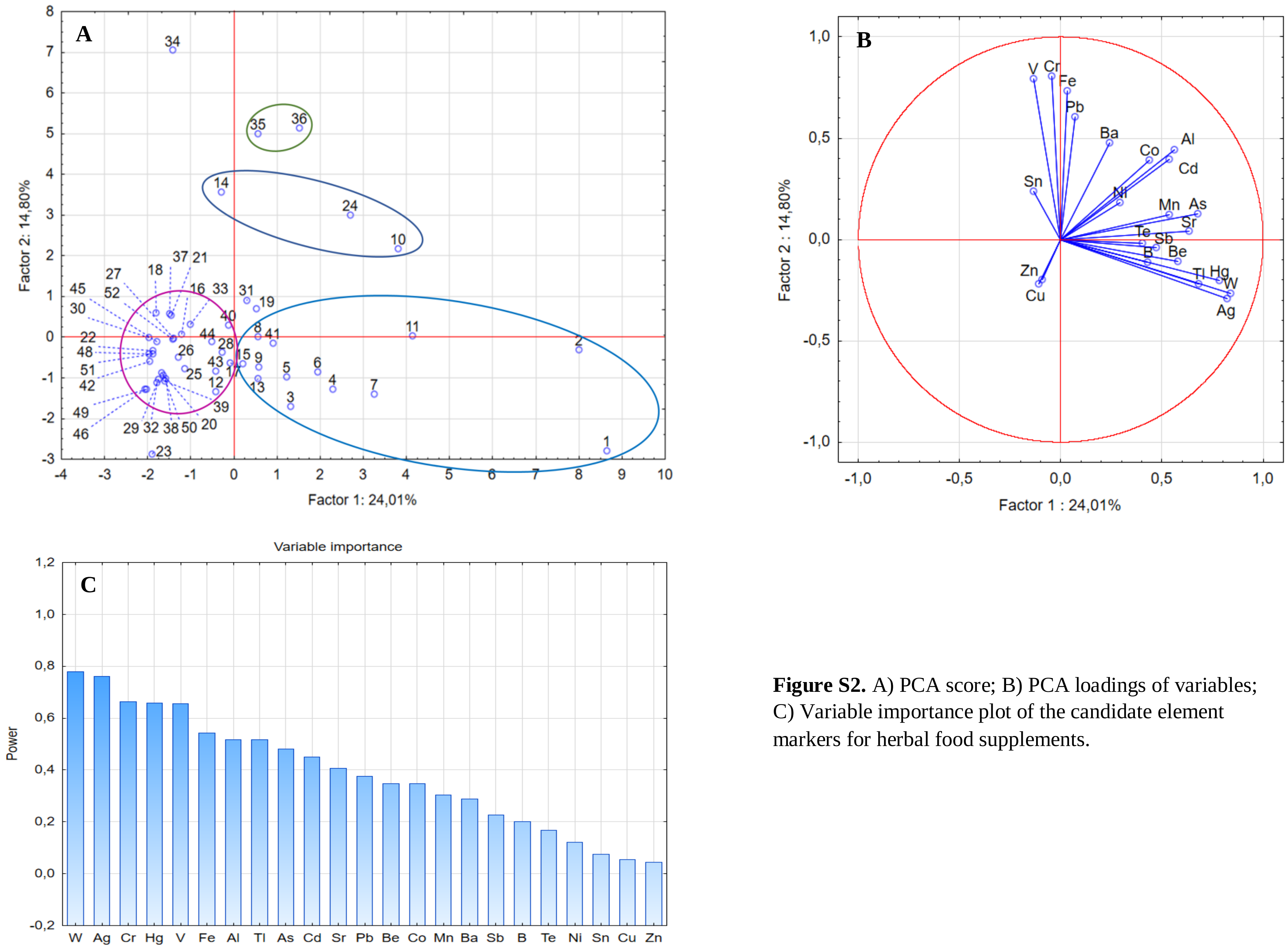

Figure S2. A) PCA score; B) PCA loadings of variables; C) Variable importance plot of the candidate element markers for herbal food supplements.

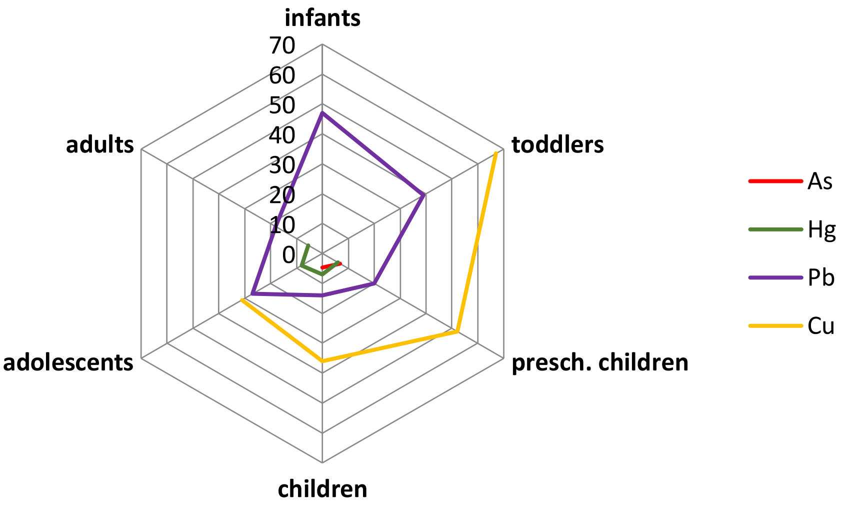

Figure S3. Maximum achieved %PDE contributions across the population groups.

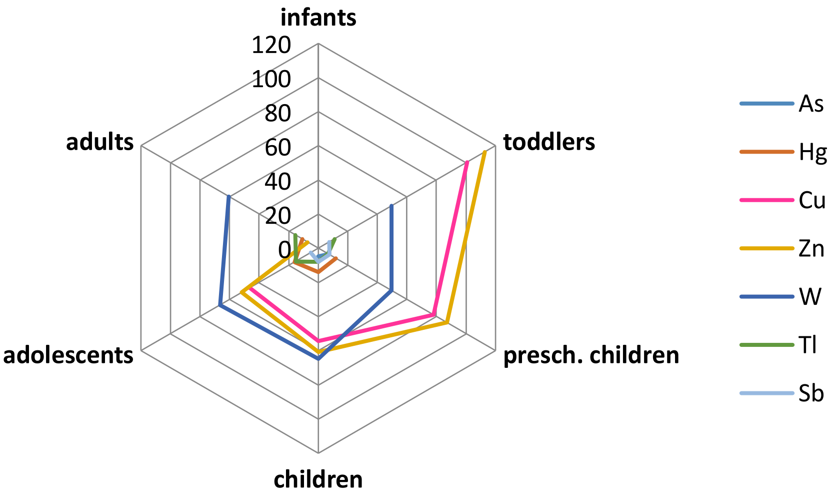

Figure S4. Maximum achieved %RfD contributions across the population groups.

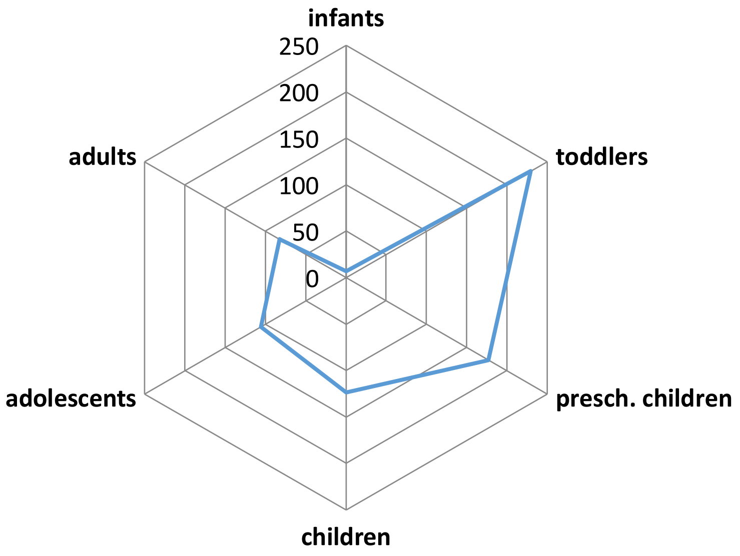

Figure S5. Maximum achieved %HI levels across the population groups.

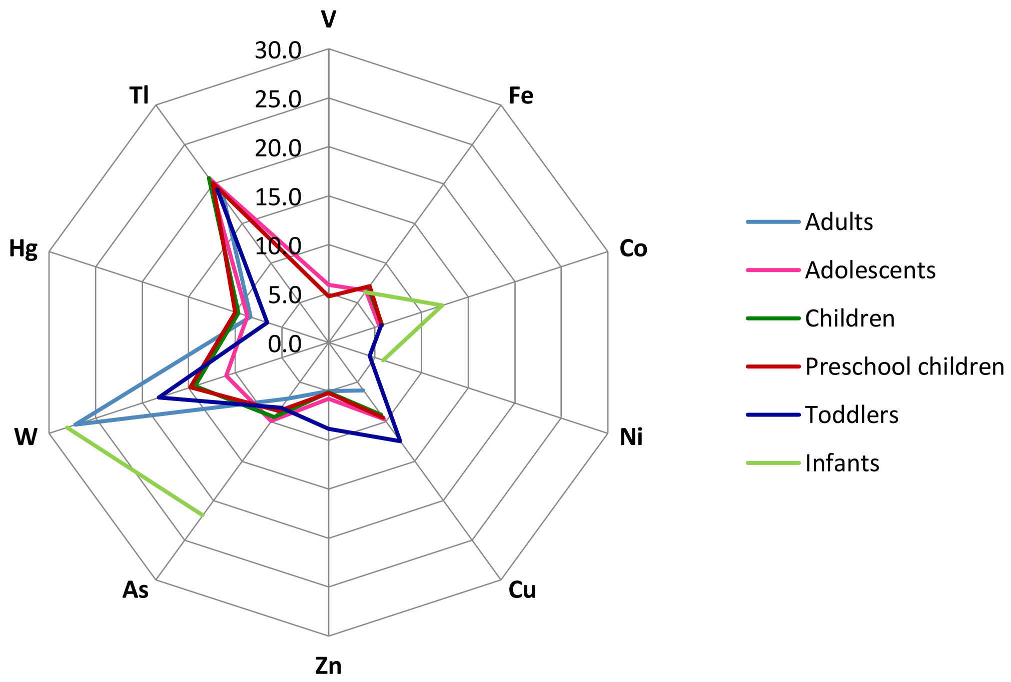

Figure S6. Mean HQ contribution (%) to HI across the population groups. Note: only elements reaching at least 5% of HI are presented.

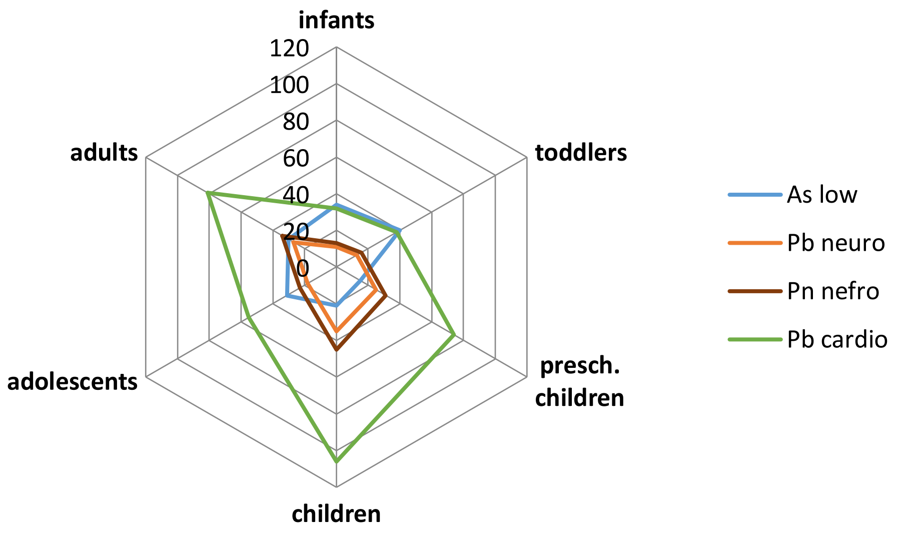

Figure S7. Minimum achieved As and Pb MOE levels across the population groups.

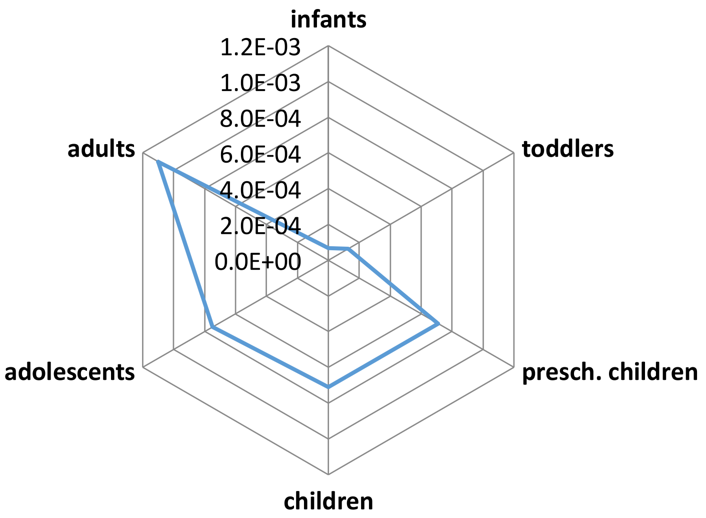

Figure S8. Maximum achieved As LCR levels across the population groups.
